# Training for outbreak response through the Global Outbreak Alert and Response Network

**DOI:** 10.1186/s12916-021-01996-5

**Published:** 2021-05-14

**Authors:** Renee Christensen, Dale Fisher, Sharon Salmon, Patrick Drury, Paul Effler

**Affiliations:** 1 World Health Organization, Geneva, Switzerland; 2 Yong Loo Lin School of Medicine, National University of Singapore, Singapore, Singapore; 3 University Medicine Cluster, National University Hospital, Singapore, Singapore; 4 World Health Organization, Regional Office for the Western Pacific, Manila, Philippines; 5 Indo-Pacific Centre for Health Security, Department of Foreign Affairs and Trade Australia, Barton, Australia; 6 School of Medicine, University of Western Australia, Perth, Australia

**Keywords:** Outbreak response, Training, Capacity building, GOARN, Scenario

## Abstract

For 20 years, the Global Outbreak Response and Alert Network (GOARN) has been a leader in the coordination of international outbreak response. On the premise that no single institution can provide all capacities required to successfully respond to a complex public health emergency or fulfil all outbreak response training needs, GOARN embarked on a capacity building journey that draws on the unique strengths of more than 250 partner institutions. Through extensive engagement and collaboration, GOARN Partners have created a bespoke, multifaceted, 3-tiered training programme which has evolved over the last 15 years and enhanced the competencies of thousands of multidisciplinary outbreak responders around the world.

**Supplementary Information:**

The online version contains supplementary material available at 10.1186/s12916-021-01996-5.

## Introduction

Successful outbreak response requires a multidisciplinary [1] cadre of professionals who can work effectively and efficiently as part of a cohesive team. The cumulative experience of the Global Outbreak Alert and Response Network’s (GOARN) extensive engagement in international public health response over the last 20 years [2] has highlighted the need to complement an individual responder’s significant level of technical expertise with additional “soft skill” competencies, such as how to communicate effectively and enhance teamwork while appreciating and respecting individual and cultural diversity. These skills are especially vital to success when deployed internationally to a public health emergency, where a responder can find themselves working closely with individuals from a variety of technical backgrounds and cultures in often highly stressful and dynamic response environments. Technical skills training for outbreak response has historically been developed and delivered by individual institutions [3], or discipline-specific public health networks such as Field Epidemiology Training Programmes [4]. Building upon the many highly regarded and longstanding technical training programmes, the World Health Organization (WHO) and other GOARN Partners from across the globe collaborated to create and implement an evolving soft-skill competency-based GOARN Capacity Building and Training Programme designed to complement existing technical skills of outbreak response experts from a wide array of disciplines including Epidemiology, Infection Prevention and Control, Case Management, Laboratory, Risk Communications and Community Engagement. Intended as an overarching training opportunity to adequately prepare an individual to perform effectively in multidisciplinary and culturally-diverse teams, this programme helps build the core capacities required for an international responder to adapt and apply their technical skills in any type of outbreak response team or field setting. Comprised of bespoke virtual and face-to-face learning experiences, the programme is founded on widely recognized best practices for public health emergency response and adult learning theory [5]. Furthermore, it is a uniquely collaborative and needs-driven training programme collectively purpose-built by many of the world’s leading public health institutions who have joined forces together specifically to strengthen the global, international multidisciplinary response capacity for outbreaks and other public health emergencies.

## Evolution of the GOARN Outbreak Response Scenario training

As the public health emergencies landscape and corresponding response needs have evolved over the last 20 years, so too has the structure and content of the GOARN Capacity Building and Training Programme. GOARN’s inaugural training effort in 2005 saw a group of outbreak responders brought together for a didactic-style course to learn about operational processes of deploying to the field, the inter-disciplinary teamwork required in an outbreak response, team leadership, ensuring team member well-being on deployment, and best practices for engaging with communities and other partners. The training was delivered in a classroom setting by WHO experts together with an international faculty of highly experienced outbreak responders drawn from across the network. After several iterations of this didactic training and the lessons learned from implementing similar classroom-style trainings, recommendations from an external evaluation of the course conducted in 2009, and experiences stemming from a Training of Trainers seminar in 2010, the GOARN course was converted into a 7-day residential scenario-driven training. The new course was a mix of didactic sessions and simulated role-play [6] with facilitated reflections shaped by a course faculty comprised of a carefully curated multidisciplinary team drawn from GOARN partners. Set in a fictious South-East Asian country, the scenario encompasses the first 6 weeks of an evolving outbreak of unknown aetiology, where participants are deployed as part of an international outbreak response team to support the local health authorities with their investigation.

**Fig. 1:**
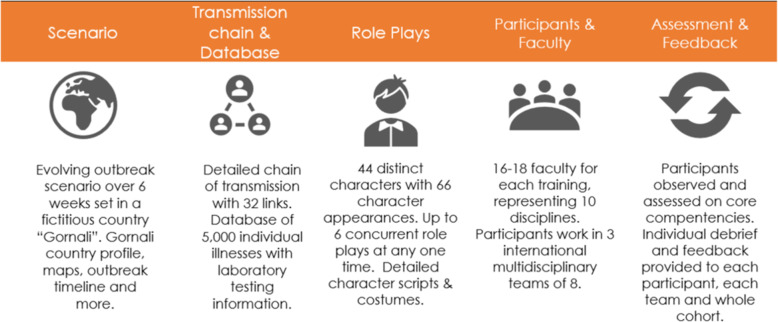
Infographic with key features of the GOARN Outbreak Response Scenario Training

The advantage of this training is the unique multidisciplinary outbreak response scenario and accompanying package of teaching and learning materials, including simulated role-plays and inter-disciplinary tasks that the participants need to complete. Throughout this highly intense training, participants work in one of three separate multidisciplinary outbreak response teams and must demonstrate competence in working effectively together to overcome challenges, allocate roles and responsibilities amongst themselves, manage their time appropriately, set priorities, and submit deliverables. The course emphasizes ensuring that the work done by each technical discipline is complementary to the other disciplines in the team while operating under increasing public health, community, and political pressure to rapidly identify the pathogen and recommend appropriate control measures. Key features of the GOARN Outbreak Response Scenario training can be seen in Fig. 1 below.

The GOARN Outbreak Response Scenario Training was launched in 2011 with overwhelmingly positive feedback from course graduates, and with increasing demand for participation by outbreak responders worldwide quickly became the network’s flagship course. However, due to the significant human and financial resources required to organize and deliver the course, as well as the need to deploy resources to the West African Ebola response (2013–2016) [7], the course was only run another 3 times between 2011 and 2015 with 24–27 participants each course.

## Lessons learned and the need to scale up access for outbreak responders to GOARN Capacity Building and Training opportunities

In the wake of the West African Ebola outbreak, GOARN endeavoured to scale up and evolve training opportunities to meet the growing demand for outbreak response capacity. After undertaking a soft-skill competency-based needs analysis and developing a corresponding competency framework [8] describing the traits and behaviours of the “ideal” GOARN outbreak responder, a new multi-faceted, multi-tiered approach for delivering a competency-based GOARN Capacity Building and Training Programme was proposed and endorsed by network partners in early 2016.

Key elements of the new programme stemmed from the original GOARN Outbreak Response Scenario Training. In order to streamline the course to be less resource-intensive, it was reduced from 7 to 5 days, with the content that was removed incorporated into other learning opportunities. Most notably the didactic content was integrated into a series of self-directed eCourses (called Tier 1) and some leadership elements were greatly enhanced and incorporated into an emerging leadership-specific training component (Tier 3).

The 5-day GOARN scenario training (Tier 2) was subsequently transformed into a full scenario-driven exercise with approximately 80% of the course in simulation, and the time “out-of-scenario” mostly dedicated to facilitated team and individual debriefs. In addition, the structure of the scenario was tightened, formative and summative assessments of learning were embedded throughout the course, and all learning objectives, injects and role plays were assessed against the GOARN Competency Framework for relevance, prioritization and the ability to evaluate participant learning and progress. For the purposes of the simulation, costumes, props and real physical locations were introduced with the use of actual WHO Country Offices, hospitals, city and field locations for the role plays. Coordination of the simulation exercise was digitized, with delivery of injects and provision of in-character feedback to participants on their deliverables using email, video and online messaging. This enables the scenario to remain dynamic, with the faculty able to add new or different injects to address key learning points as necessary, and the participants to follow the best practices for the learning and assessment cycle. In addition, a rigorous participant application and selection process was introduced, including review of resumes and letters of motivation and institutional support for future deployment. Interviews addressing the core GOARN Competencies [8] are conducted, with final selections of participants being made to ensure appropriate diversity of technical disciplines, gender and global geographic representation (with typically 25–50% of participants representing the region in which the training is being hosted). The likelihood of the individual deploying on an outbreak response in the future is also assessed, considering the candidates’ application, the role in their organization and the letter of institutional support for future deployment.

With a corresponding scale-up in the roster of prepared and available multidisciplinary and multilingual faculty, the frequency of delivering the scenario-driven simulation exercise increased by almost 4-fold. As the training was conducted more frequently, word its benefits spread amongst the global outbreak community. Despite increasing the number of participant places to 144 across 6 courses between 2016 and 2019, with over 900 applications received during this time it was still not possible to meet demand. In an effort to scale up and deliver the courses even more frequently, GOARN Partners continue to consider ways to ensure senior outbreak response experts across all public health disciplines who are also trained in pedagogic best practices and adult learning theories are periodically available to facilitate the course and to engage professionals with relevant simulation exercise coordination experience to lead course organization and delivery. GOARN Partners are also exploring possibilities to “franchise” the training to one or more partners so they can deliver the course independently, in multiple languages to reach a much wider audience. While principles for franchising have been developed and approved by GOARN Partners, which detail the required steps for preparing faculty and delivering and evaluating the training, challenges still exist to any one institution being able to effectively deliver the course on their own. This is partly due to the high resource requirements involved and the fact that no single institution has the required multi-disciplinary outbreak response expertise to do so—the very gap that prompted the course to be developed by GOARN in the first place. As such, GOARN is examining opportunities for interested and capacitated Partners across different public health disciplines, possibly from the same country or the same region, to work together to co-host and co-deliver the Outbreak Response Scenario Training going forward.

## Development of the three-tiered competency-based programme

**Fig. 2:**
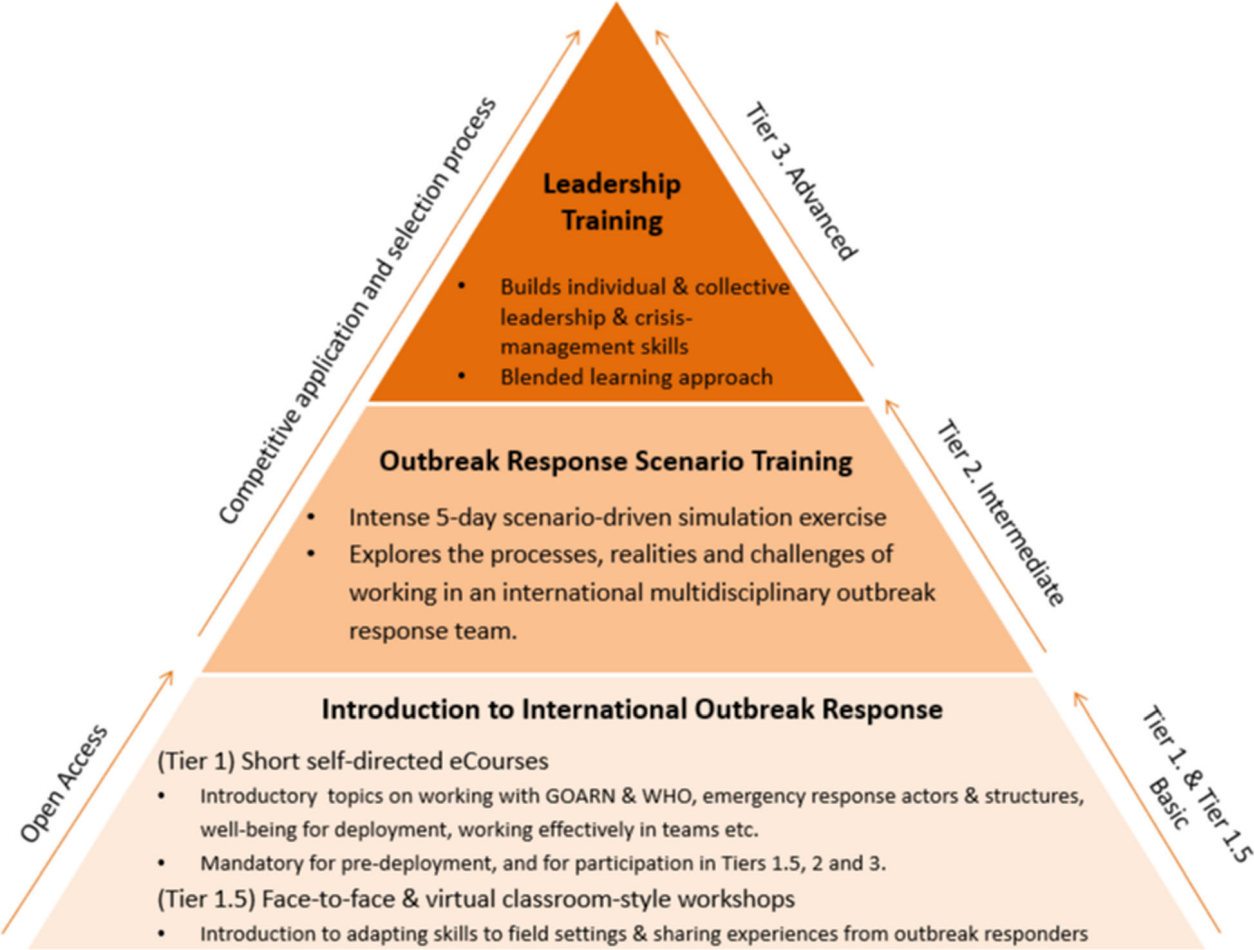
Illustration of the 3-tiered GOARN Capacity Building and Training Programme

The GOARN Competency Framework was mapped against the capacity needs assessment to inform the scope and design of content for what subsequently became the 3 tiers of the new GOARN Capacity Building and Training Programme, as illustrated in Fig. 2 below. Refer to Additional File 1 for details of the GOARN Competency Model.

**Fig. 3:**
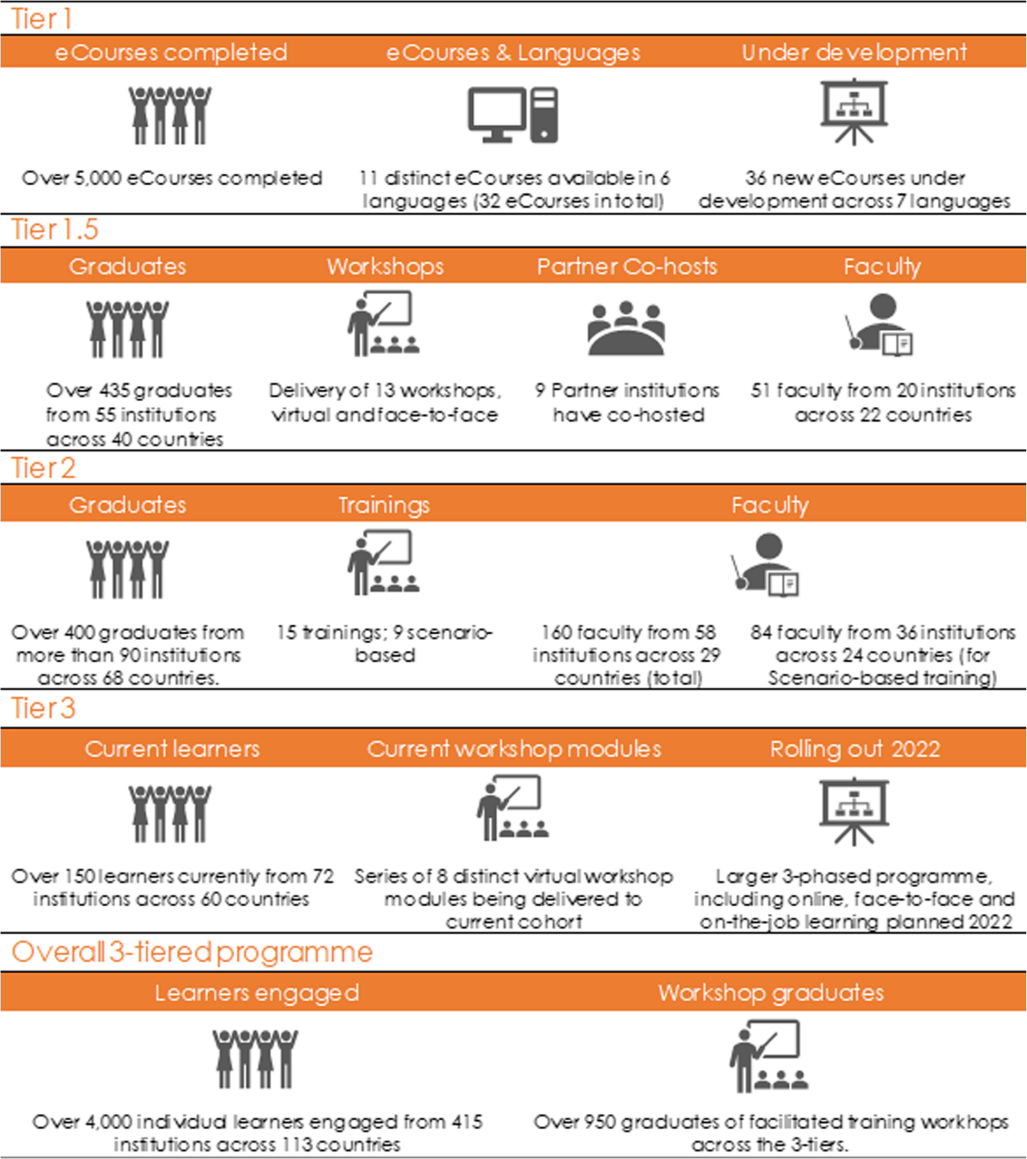
Infographic with key data across each tier of the GOARN Capacity Building and Training Programme

The first tier of the programme introduces the basic essentials for pre-deployment through a series of open-access online self-directed eCourses which can be undertaken by the learner at their own pace, in one or more sittings, as well as more advanced 1–2-day classroom-based workshops (known as Tier 1.5), which take place on request, often as part of in-service public health courses for Field Epidemiology Training Programmes [4]. Similar discipline-specific, in-service training approaches have also been undertaken, for example with Infection Prevention and Control Networks and work with other disciplines being explored. As noted above, Tier 2 is the immersive 5-day outbreak response scenario-driven simulation exercise training, which addresses the realities and challenges of working in an international multidisciplinary outbreak response team. Tier 3 comprises specialized outbreak response leadership training for experienced responders which has been designed to build the individual and collective leadership and crisis management skills so deployees are well-prepared to serve as influential and trusted leaders during public health emergencies. Completion of select Tier 1 courses is a prerequisite to undertake the Tier 1.5, Tier 2, and Tier 3 trainings. GOARN has also built a virtual training platform [9] which hosts all the online courses and maintains the learning records of graduates across all tiers of the programme. The GOARN virtual training platform is also linked to the GOARN operational deployment platform, enabling visibility of these records for persons applying for deployment, with respect to completion of mandatory (Tier 1) and desirable (all other) GOARN Training courses prior to deployment. There is an option to mark a Tier 1 course completed by “*equivalence*” for instances when deployees have completed similar or more detailed eCourses available elsewhere and it is deemed unnecessary for them to repeat the training again with GOARN. Key data across each tier of the programme, including numbers and types of courses, graduates and faculty, can be seen in Fig. 3 below.

**Fig. 4:**
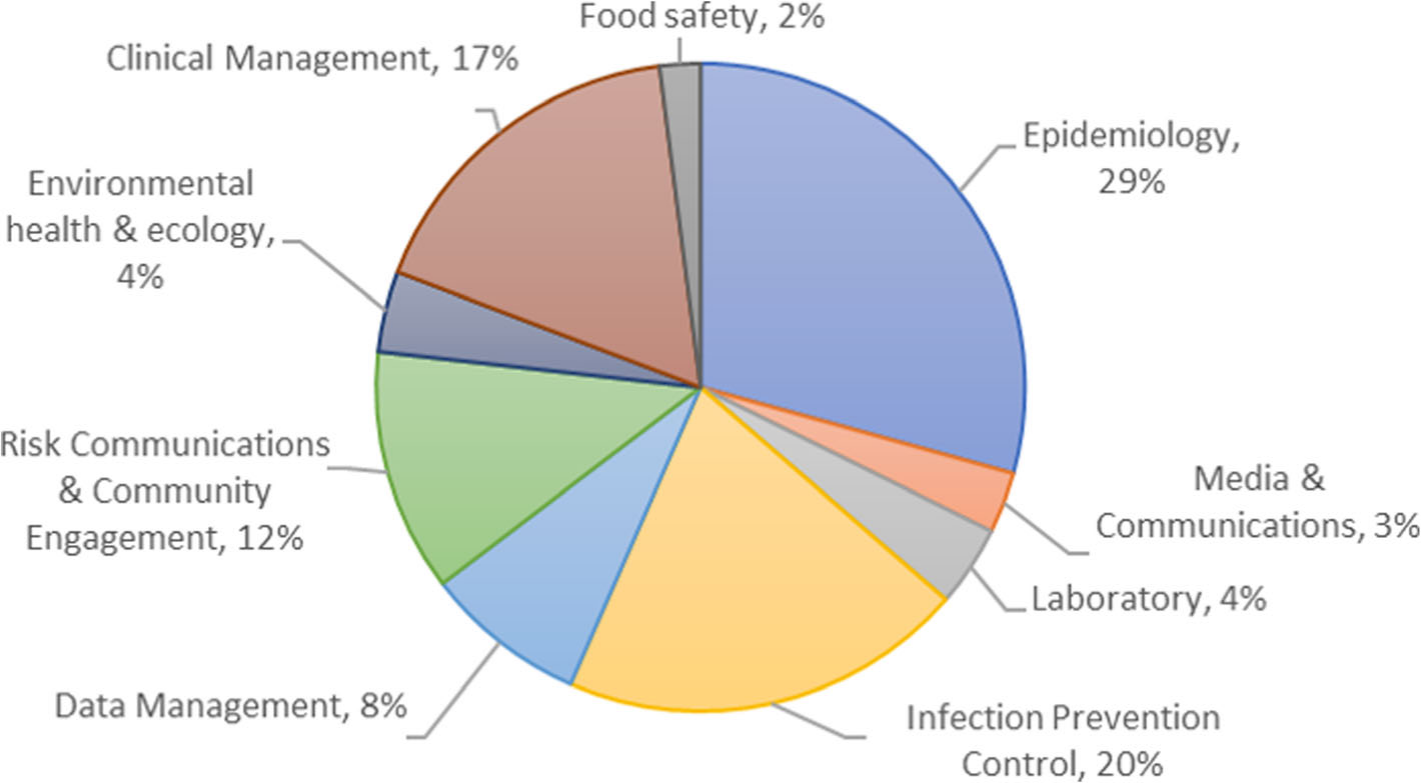
GOARN Capacity Building and Training Programme: Breakdown of learners by technical discipline

**Fig. 5:**
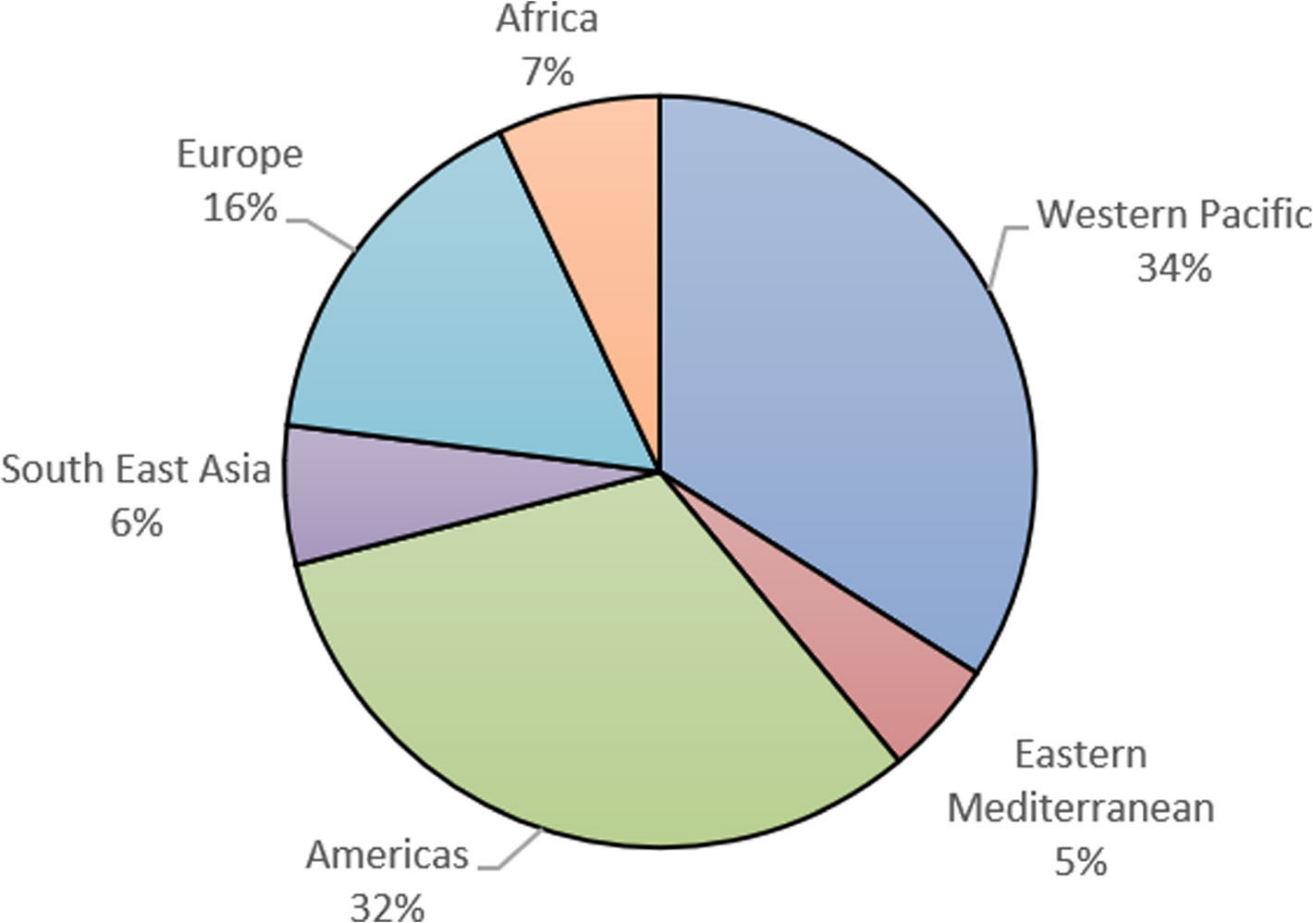
GOARN Capacity Building and Training Programme: Breakdown of learners by WHO Region

To ensure consistency in quality and relevance—with the various courses remaining needs-driven and assuring that effective learning is not only taking place but is being applied in the relevant channels of work—an evaluation framework [10] is embedded at the core of the GOARN Capacity Building and Training Programme. Applying the Kirkpatrick Model [11], all GOARN courses include formal summative assessments of learning, with the facilitated courses also including formative learning checks and participant evaluations of the perceived value of the course, and how they anticipate the application of what they have learned in their usual workplace as well as when working in an outbreak response. Refer to Additional File 2 for details of the GOARN Training Programme Evaluation Framework. All evaluation feedback is used to continuously revise and improve future courses. Furthermore, GOARN has recently undertaken the development of a Monitoring and Evaluation framework for all network activities that will include standardized debriefs post-mission to assess perceived preparedness for the deployment and identify remaining gaps in training. In addition, GOARN is examining ways to correlate participation in training with subsequent involvement in GOARN deployments. The Programme has grown substantially over the last 4 years, offering new and diverse learning opportunities through both remote and in-person methods, thus greatly increasing the programme’s reach and ultimately the pool of public health specialists trained for multidisciplinary outbreak response. While participation in online learning opportunities has greatly enhanced engagement with learners in some parts of the world (notably the Western Pacific and Americas, as depicted in Fig. 5), the lower engagement in Tier 1 of participants from Africa, Eastern Mediterranean and South East Asia may in part be due to language availability and challenges in accessing reliable internet and suitable digital devices. As such, GOARN is continuing to translate courses into additional languages and explore possibilities for adapting and creating learning opportunities with low bandwidth and off-line capabilities to increase engagement globally. Refer to Figs. 4 and 5 respectively for a relative breakdown of programme learners by technical discipline and by WHO Region.

## The partners driving the programme to excellence

The GOARN Capacity Building and Training Programme is designed *for* and in collaboration *with* outbreak response Partners [12]. When the GOARN Capacity Building and Training Network was launched in early 2016, 38 of the world’s leading academic and operational Institutions specializing in outbreak response, committed to actively engage in the collaborative design, development, delivery, monitoring and evaluation of courses across all tiers. For any new courses to be created, sub-working groups were established to steer the scope and oversee the development process, with each sub-working group co-led between the GOARN Secretariat and a nominated Partner with specific relevant expertise in the course subject matter. At the time of writing, the GOARN Capacity Building and Training Network has seen 49 institutional representations across 4 established sub-working groups. The unprecedented levels of collaboration and engagement by partners have served to dramatically enhance the quality, relevance, scalability, exposure and accessibility of the various capacity building opportunities the programme offers to both current and future outbreak response professionals. The significant in-kind contributions from partners reflect the perceived value of this programme not only to the individual outbreak responders, but also to the institutions engaged in educating and deploying them. Such contributions include staff time in working groups, providing advice as subject matter experts, sharing of content, institution funded-travel to face-to-face events, translation of content into additional languages, co-hosting of workshops and the provision of venues and equipment. For example, 11 partner institutions generously provided in-kind support for the translation of eCourses into 6 additional languages (including French, Spanish, Arabic, Portuguese, Chinese and Russian), resulting in 108 h of new learning opportunities for non-anglophone participants.

## What next?

The collaborative process of building and implementing the GOARN Capacity Building and Training Programme represents a pivotal step towards creating high-quality, accountable and interoperable learning opportunities for international outbreak responders. The COVID-19 Pandemic has further demonstrated the critical need for a highly trained multi-disciplinary human response capacity for public health emergencies [13]. Partner institutions of the GOARN Capacity Building and Training Network have stepped up collaborative efforts to meet COVID-19 learning needs, communicating frequently via an online platform to enhance coordination, identify shared priorities, streamline access to new capacity building initiatives and foster large-scale delivery while reducing duplication and redundancy. The GOARN Capacity Building and Training Programme remains dynamic, ensuring flexibility and adaptability of the courses to meet evolving learning needs and delivery methods. To meet COVID-19 training response demands and innovating with the latest in available learning technologies, much of the programme continues to turn digital, rapidly increasing the number of available self-directed eCourses and diversifying the content to address priority technical training needs (Tier 1). Courses that were previously only delivered in-person are now also being conducted virtually with increased frequency and in multiple languages, resulting in distinct packages available for both virtual and face-to-face formats (Tier 1.5 and Tier 3). Features of learning through gamification [14] are being explored, with options for adapting key courses such as the Outbreak Response Scenario Training (Tier 2) with use of virtual [15], augmented and mixed realities to enhance the learning experience. The COVID-19 pandemic has driven increased attention and engagement by partners in the GOARN Capacity Building and Training Network to support training activities useful at a national level. Opportunities to evolve relevant courses into existing professional in-service learning programmes, through franchising and possible accreditation, will serve to further broaden engagement of the overall global public health emergencies workforce to build capacity among local, national and international outbreak responders.

The GOARN Capacity Building and Training Programme remains committed to engaging with leading outbreak response professionals and institutional partners from around the globe, and across all public health disciplines, to grow its innovative, state-of-the-art, training program and strengthen outbreak response capacity worldwide.

## Supplementary Information

**Additional file 1.** GOARN Steering Committee Working Group for Training. GOARN Competency Model. 2017. This document presents the GOARN Competency Model, detailing the approach and methodology for its development and suggested application for the GOARN Capacity Building and Training Programme.

**Additional file 2.** GOARN Steering Committee Working Group for Training. GOARN Training Programme Evaluation Framework. 2017. This document presents the GOARN Training Programme Evaluation Framework, detailing the approach and design utilizing the Kirkpatrick model for training evaluation and suggested application for the GOARN Capacity Building and Training Programme. This document includes both suggested means for Level 1, 2 and 3 training evaluation across all Tiers of the Training Programme as well as sample logical frameworks for monitoring and evaluation of programme overall.

## Data Availability

The datasets used and/or analysed during the current study are available from the corresponding author on reasonable request.

## Abbreviations

GOARN: Global Outbreak Alert and Response Network
WHO: World Health Organization
