# Supplementary material for: Training for outbreak response through the Global Outbreak Alert and Response Network
Source: BMC Med. 2021 May 14;19:123. doi: 10.1186/s12916-021-01996-5 (PMC8118749; doi:10.1186/s12916-021-01996-5)
Supplement: Supplementary file 1 — Additional file 1. GOARN Steering Committee Working Group for Training. GOARN Competency Model. 2017. This document presents the GOARN Competency Model, detailing the approach and methodology for its development and suggested application for the GOARN Capacity Building and Training Programme. [file 12916_2021_1996_MOESM1_ESM.pdf]

Document produced by the GOARN Steering Committee Working Group for Training and endorsed by the GOARN Training Network in 2017.

# GOARN Competency Model:
# For team member and team leader deployees

## What are competencies?

The term "competency" refers to a combination of skills, attributes and behaviours that are directly related to successful performance on the job". Defining competencies is important as they help clarify expectations, and undertake more focused nomination and recruitment, development and training planning. Competencies also serve to provide a sound basis for setting and monitoring consistent and objective performance standards.

## The GOARN Competency Model

The GOARN Competencies are categorized as *Core* competencies that describe the skills, attributes and behaviours considered important for all GOARN deployees, regardless of their job function or level of seniority, and also *Managerial* or *Leadership* Competencies which are considered essential for individuals deployed as team leaders or field coordinators with specific managerial or supervisory responsibilities. Each competency is supported by a definition, GOARN context description and a series of desired behavioral indicators.

At present GOARN is supported by the WHO human resource programme for hiring and engaging the staff of partner organisations made available to participate in Network deployments and activities. Accordingly, it was decided to use the WHO Global Competency Model as an overarching basis to which additional skills, behaviours and attributes were added to meet the particular qualities relevant for GOARN team member and team leader deployees who will be working specifically in outbreak public health emergency environments. To ensure consistency and interoperability across the emergency and outbreak communities, for all relevant identified GOARN qualities not explicitly articulated in the WHO Global Competency Model, where possible, a competency behavioural indicator from an existing and widely accepted competency framework, was identified and inserted, and in some cases with language tailored to the GOARN context[1].

## How to use the GOARN competency model

It is suggested for the GOARN Competency model to be considered for use in the following ways:

- To both review existing and create new training courses that ensure learning outcomes build and measure the desired and select qualities and behavioural indicators of team member and team leader profiles.
- GOARN Partners are encouraged to use the GOARN Competency Model as a reference for their nomination of potential suitable deployees.
- For overall quality assurance and accountability of the GOARN network in surge deployments.

This GOARN Competency Model and the many ways it can and should be encouraged for use, represents a pivotal step towards building and ensuring quality and accountable training and capacity development opportunities for GOARN members, and supporting efforts to unify and harmonize these efforts across GOARN, WHO and the rest of the public health emergency community.

[1] These existing and widely accepted frameworks include Inter-Agency Standing Committee Humanitarian Coordination Competencies (IASC, 2011), Consortium of British Humanitarian Agencies (CBHA) Core Humanitarian Competency Framework (CBHA, 2010), United Nations Competencies Framework (UN Office for Human Resource Management, 2010)

## Core Competencies for all GOARN Deployees

### 1. Communicating in a credible and effective way

**Definition:** Expresses oneself clearly in conversations and interactions with others; listens actively. Produces effective written communications. Ensures that information is shared.

**GOARN context:** GOARN deployees need to be able to speak and write clearly and concisely, and actively listen to others – including team members, partners and crisis-affected people - in order to appropriately interpret their message, discover new and different perspectives and respond appropriately. GOARN deployees may be faced with challenging interactions, whereby strong communication and negotiation skills will assist in bringing opposing positions to an acceptable compromise. GOARN deployees will also sometimes lead, and regularly participate in meetings in which they need to be able to tailor their language, tone, style and format to the meet the needs of their audience, projecting credibility and influencing or persuading others of a course of action. GOARN deployees may also need to make presentations and draft situation updates and reports, and should be confident in public speaking and applying these various communication skills in both their speaking and writing.

| Behavioural Indicators | |
|---|---|
| 1. | Encourages others to share their views; takes time to understand and consider these views |
| 2. | Actively listens to new and different perspectives and experiences of affected people, stakeholders and team members |
| 3. | Speaks and writes clearly, adapting communication style and content so they are appropriate to the needs of the intended audience |
| 4. | Conveys information and opinions in a structured and credible way |
| 5. | Can effectively influence or persuade others of a course of action |
| 6. | Makes presentations and undertakes public speaking with skill and confidence |
| 7. | Ensures that messages have been heard and understood |
| 8. | Keeps others informed of key and relevant issues |

### 2. Knowing and Managing Yourself

**Definition:** Manages ambiguity and pressure in a self-reflective way. Uses criticism as a development opportunity. Seeks opportunities for continuous learning and professional growth.

**GOARN context:** GOARN deployees might encounter situations in which there is ambiguity regarding roles, responsibilities and workplans, and as such they will need to remain positive and productively take considered initiative for undertaking tasks when work is not explicitly assigned. While on assignment, GOARN deployees may also be tasked with assignments that could be considered below their level of qualification or expertise, and will need to manage their own expectations for the nature, level and quantity of theirs – and their colleagues – related work outputs. GOARN deployees should exhibit willingness to learn from mistakes, and seek feedback in order to continually improve their performance. GOARN deployees will also often be working in highly stressful environments, and so need to take responsibility for their own physical and mental well-being, and take precautions to ensure personal safety and security.

| Behavioural Indicators | |
|---|---|
| 1. | Works productively in an environment where clear information or direction is not always available |
| 2. | Remains productive when under pressure |
| 3. | Stays positive in the face of challenges and recovers quickly from setbacks |
| 4. | Uses constructive criticism to improve performance |
| 5. | Shows willingness to learn from previous experience and mistakes, and applies lessons to improve performance |
| 6. | Seeks feedback to improve skills, knowledge and performance |
| 7. | Promotes well-being and a ‘duty of care’ culture |
| 8. | Reduce vulnerability by complying with safety and security protocols set by your organisation and adapt it to the local context |

### 3. Producing Results

**Definition:** Produces and delivers quality results. Is action oriented and committed to achieving outcomes.

**GOARN context**: As technical experts, GOARN deployees should demonstrate the highest level of professional competency of their technical speciality, exhibiting a service-oriented approach that ensures the execution of their role is proactive and accountable. For professional competence, GOARN deployees need to take responsibility for the efficiency and quality of their work, committing to keeping abreast of the latest developments relevant to their field. GOARN deployees need to have the skills to develop clear work goals that are consistent with agreed strategies, identify priority activities, foresee risk, manage time effectively and allow for contingencies and adjust priorities as needed. In field environments, there is often a high turn-over of staff, and as such GOARN deployees need to effectively see their work through to completion, or if time does not permit, ensure there is an adequate handover. Working in an outbreak or humanitarian response also requires frequent updates of varying sources and types of information, which require a GOARN deployee to have the skills to

effectively analyse and interpret data to ensure they are working in an informed environment. GOARN deployees on mission will also be faced with situations in which they will need to exercise critical judgement in making informed, and often rapid, decisions as both an individual and as part of a group, including decisions related to the escalation of any unresolved issues to higher authorities (such as the local WHO Representative of WHO Headquarters).

| Behavioural Indicators |
|---|
| 1. Demonstrates a systematic and efficient approach to work |
| 2. Produces high-quality results and workable solutions that meet client needs |
| 3. Demonstrates professional competence and mastery of subject matter |
| 4. Be aware of internal and external influences that affect your performance |
| 5. Foresees risks and allows for contingencies when planning |
| 6. Monitors own progress against objectives and takes any corrective actions necessary |
| 7. Monitors costs and seeks to use the most cost-effective methods |
| 8. Acts without being prompted and makes things happen; handles problems effectively |
| 9. Takes responsibility for own work |
| 10. Sees tasks through to completion |
| 11. Is conscientious and efficient in meeting commitments, observing deadlines and achieving results |
| 12. Uses time efficiently |
| 13. Makes rational judgements from the available information and analysis |
| 14. Makes prompt, clear decisions which may involve tough choices or considered risks |
| 15. Demonstrates understanding of when a decision can be taken and when to involve others |

**4. Moving forward in a changing environment**

**Definition**: Is open to and proposes new approaches and ideas. Adapts and responds positively to change.

**GOARN context**: GOARN deployees will sometimes be sent to locations and situations in which the environment and circumstances can change rapidly, and as such they need to possess a multi-faceted ability to cope and adapt with these dynamic and often stressful situations. The environments in which the GOARN deployees are based will vary greatly, with some last minute changes of location possible as well insecurity concerns resulting in access or transportation restrictions or possible reassignment. GOARN deployees also need to manage expectations of their possible working and residential environments, and travel prepared, as they will often be sent to locations that lack many modern facilities and amenities such as internet, power and running water. GOARN deployees will also often be joining an on-going outbreak or humanitarian response, and will need to be open and flexible to different ways of working and operating, exhibiting willingness to compromise and accept other perspectives when necessary.

| Behavioural Indicators |
|---|
| 1. Is receptive to new ideas and working methods |
| 2. Actively supports change initiatives |
| 3. Recognises opportunities for improvement and proposes workable solutions |
| 4. Actively seeks to apply new methods and technologies to improve work processes |
| 5. Understands applicability and limitations of technology to the work of the Office |
| 6. Adapts readily and efficiently to changing priorities and demands |
| 7. Demonstrate flexibility to adapt in situations of rapid change, always informed by a focus on affected people |
| 8. Remain constructive and positive under stress to be able to tolerate difficult and challenging environment |

**5. Fostering integration and teamwork**

**Definition:** Develops and promotes effective relationships with colleagues and team members. Deals constructively with conflicts.

**GOARN context:** GOARN deployees will very often be sent to work within a team environment, and as such they need to be able to seamlessly join and work with a diverse range of individuals and teams, often without knowing any of the team members prior to arrival and frequently as the only "outsider". GOARN deployees need to possess the capacity to integrate quickly, build trust with fellow team members, and often individuals from other units or organisations, swiftly establishing ways to contribute to positive, productive and effective ways of working together.

| Behavioural Indicators |
|---|
| 1. Works collaboratively with team members to achieve results |
| 2. Encourages co-operation and builds rapport among fellow team members |
| 3. Supports and acts in accordance with team decisions |
| 4. Accepts joint responsibility for team's successes and shortcomings |
| 5. Identifies conflict early and supports actions to facilitate its resolution |
| 6. Solicits input by genuinely valuing others' ideas and expertise; is willing to learn from others |
| 7. Show awareness of your own strengths and limitations and its impact on others |
| 8. Demonstrate understanding of your skills and how they complement those of others to build team effectiveness |

9. Share useful information and knowledge with colleagues, partners, and crisis-affected people as and when appropriate
10. Establish agreed ways of working at a distance with partners and staff

### 6. Respecting and promoting individual and cultural differences

**Definition:** Demonstrates the ability to work constructively with people of all backgrounds and orientations. Respects differences and ensures that all can contribute.

**GOARN context**: GOARN deployees will be working with diverse groups of people, and will need to work seamlessly and effectively with both colleagues and affected populations of all backgrounds, cultures, religions, genders and ages. As such, GOARN deployees need to exhibit the skills and attitudinal competencies for treating all individuals with dignity and respect and without any form of discrimination, and also recognize the differing needs, skills, capacities and experiences of others and utilise them in their work. GOARN deployees need to be able to see things from the perspective of others, including the affected population, organisational partners and peers, in order to establish and maintain productive partnerships, trust and response, and to identify needs and appropriate solutions.

Behavioural Indicators

1. Relates and works well with people of different cultures, gender and backgrounds
2. Examines own behaviour and attitudes to avoid stereotypical responses
3. Considers issues from the perspective of others
4. Takes into account the needs, skills, capacities, and experiences of affected people and apply these in the response
5. Draws on diversity of skills, backgrounds and knowledge of people to achieve more effective results
6. Understands and respects cultural and gender issues and applies this to daily work and decision making
7. Establishes and maintains productive partnerships with local authorities and populations by gaining their trust and respect

### 7. Setting an example

**Definition:** Acts within the Organisations professional, ethical and legal boundaries and encourages others to adhere to these. Behaves consistently in accordance with clear personal ethics and values.

**GOARN context:** GOARN deployees are contracted under WHO, and as such they need to appreciate and abide by WHO's professional, ethical and legal boundaries, as well as the GOARN Code of Conduct. As GOARN deployees will often be sent to a declared humanitarian emergency, they need to demonstrate understanding and adherence to the humanitarian principles, and possess sound knowledge of humanitarian and public health architecture including relevant coordination structures, stakeholders and the humanitarian programme cycle.

Behavioural Indicators

1. Understand and behaves in accordance with WHO's professional ethical and legal framework (*and GOARN General Principles*)
2. Demonstrates consistency between expressed principles and behaviours
3. Is transparent in dealings with others
4. Takes action when others behave in an unprofessional or unethical manner
5. Maintains confidentiality and treats sensitive information with discretion
6. Stands by own decisions or actions and takes responsibility for them
7. Maintain ethical and professional behaviour in accordance with GOARN and the United Nations codes of conduct
8. Apply understanding of the political and cultural context and underlying causes of the crisis
9. Demonstrate understanding of your role and that of your organization and others within the response to the outbreak or public health emergency

### 8. Building and promoting partnerships across the organization and beyond

**Definition:** Develops and strengthens internal and external partnerships that can provide information, assistance and support to the organisation. Identifies and uses synergies across the organization and with external partners.

**GOARN context:** GOARN deployees are expected to build and maintain professional synergistic relationships with stakeholders involved in the response, in order to benefit their work through access, information and provision of support, which in turn will benefit the affected populations. GOARN deployees will often be expected to hold and lead regular meetings with partners, collecting and sharing information to foster collaboration and promoting synergies.

Behavioural Indicators

1. Seeks to understand and promote synergies between the work produced in WHO, *GOARN,* and the work of external partners to improve the organisational success
2. Builds and maintains mutually beneficial work relationships and alliances inside and outside the organisation *(GOARN and WHO)*
3. Encourages and assists others in building networks to improve results
4. Creates opportunities for promoting synergies inside and outside the organization *(GOARN and WHO)* to improve outcomes

## Competencies for GOARN responders deployed as Team Leaders and Field Coordinators

### 9. Creating an empowering and motivating environment

**Definition:** Guides and motivates staff towards meeting challenges and achieving objectives. Promotes ownership and responsibility for desired outcomes at all levels.

**GOARN context:** GOARN responders deployed as Team Leaders or Field Coordinators are expected to ensure that roles and responsibilities of all team members are clear, and work to motivate, encourage and support each team member, ensuring they are enabled to produce quality and relevant work. They further need to be able to prioritise, organise, re-delegate and manage their teams, adapting their management style to support and meet the needs of all individual team members, while also fostering integration and teamwork to produce results and meet overall team objectives.

Behavioural Indicators

1. Provides staff with clear direction and support in meeting their objectives
2. Ensures that roles, responsibilities and reporting lines are clearly defined, understood and accepted
3. Delegates work appropriately to staff, providing them with the necessary support to meet their objectives
4. Shows confidence in staff and encourages initiative
5. Provides staff with regular feedback, recognises good performance and addresses performance issues
6. Motivates staff to achieve individual and team goals

### 10. Ensuring the effective use of resources

**Definition:** Identifies priorities in accordance with WHO's (*and GOARN's)* strategic directions. Develops and implements action plans, organizes the necessary resources and monitors outcomes.

**GOARN context:** GOARN responders deployed as team leaders or field coordinators are responsible for ensuring the effective and considered use of resources within their team assignments – including financial and human – at all times, in all circumstances, according to WHO staff and GOARN Guiding Principles expectations.

Team leaders are expected to assume continued responsibility for the identification and monitoring of the necessary human, logistical and financial resources and security needs throughout the response.

Behavioural Indicators

1. Develops plans into clearly defined objectives that take into account of changing circumstances
2. Identifies priorities and defines realistic objectives and timelines
3. Identifies, organizes and effectively manages the financial and human resources needed to achieve results
4. Is able to quickly re-allocate resources and reset priorities in response to unexpected events
5. Establishes measures to monitor resources and progress of activities as planned
6. Monitors costs and seeks to use the most cost-effective methods

### 11. Promoting innovation and organizational learning

**Definition:** Invigorates the Organization by building a culture that encourages learning and development. Sponsors innovative approaches and solutions

**GOARN context:** GOARN responders deployed as Field Coordinators or Team Leaders are responsible for positioning their team for success. These GOARN profiles should work to empower and encourage GOARN partners and team embers to share their knowledge and experiences in order to learn from each other and improve both their individual and overall work outcomes.

Behavioural Indicators

1. Drives change and improvement, continually searching for new ways to position the Organization (*GOARN, WHO and mission team*) for success
2. Encourages others to be innovative to improve outcomes
3. Ensures that knowledge and learning is shared across the Organization (*GOARN, WHO and mission team)*; encourages staff to learn from each other
4. Uses feedback to find ways to increase organizational effectiveness (*GOARN, WHO and mission team)*
5. Pushes a sense of responsibility and empowerment down the Organization (*GOARN, WHO and mission team)*
6. Creates opportunities for learning and development throughout the Organization (*GOARN, WHO and mission team)*
