# Supplementary material for: Training for outbreak response through the Global Outbreak Alert and Response Network
Source: BMC Med. 2021 May 14;19:123. doi: 10.1186/s12916-021-01996-5 (PMC8118749; doi:10.1186/s12916-021-01996-5)
Supplement: Supplementary file 2 — Additional file 2. GOARN Steering Committee Working Group for Training. GOARN Training Programme Evaluation Framework. 2017. This document presents the GOARN Training Programme Evaluation Framework, detailing the approach and design utilizing the Kirkpatrick model for training evaluation and suggested application for the GOARN Capacity Building and Training Programme. This document includes both suggested means for Level 1, 2 and 3 training evaluation across all Tiers of the Training Programme as well as sample logical frameworks for monitoring and evaluation of programme overall. [file 12916_2021_1996_MOESM2_ESM.pdf]

Document produced by the GOARN Steering Committee Working Group for Training and endorsed by the GOARN Training Network in 2017.

GOARN
Global Outbreak Alert and Response Network

# GOARN Training Programme

# Evaluation Framework

The evaluation framework proposed in this document corresponds to the training programmes outlined in the three tiers of the GOARN Training Programme Concept note[1].

## Why evaluate training?

Effective evaluation and assessment of learning and training activities are essential to the success of any skills development programme. Without thorough and appropriate evaluation, there is no way of knowing if the training or learning activity was successful or relevant. Evaluation measures the effectiveness of a programme, and is needed to ensure that learning outcomes are appropriate and have indeed been achieved, and to justify an added value of the program in question. It enables feedback on how the program was received by participants, the extent of their learning and retention, the appropriateness and effectiveness of the instructional process, the impact of programme on the participants and their organisations and identifies areas that are either missing or needing revision.

Kirkpatrick's three key reasons for evaluating training

1. To justify the existence and budget of training department by showing how it contributes to the organisations objectives and goals
2. To decide whether to continue or discontinue training programmes
3. To gain information on how to improve future training programmes

Box 1. Reasons to evaluate training, according to renowned training evaluator Donald L. Kirkpatrick[2]

## Evaluation and the training cycle

The process of analysing training needs, designing, developing, delivering and evaluating training or development courses is a cyclical process, as outlined in Figure 1. While formal program evaluation is a concrete step in this cycle, it is important to keep in mind the ways in which you will be able to effectively measure the learning that the programme is being designed to build throughout the cycle, and to continually evaluate learning as it happens. This kind of "formative" evaluation helps to ensure that the training has been designed, developed and delivered with great thought, analysis and rationale at each step and determines what needs to change in the training course plan and delivery to ensure it is most effective for participants[3].

The first step of having any kind of meaningful learning evaluation is to have the training or learning experience founded on concrete and measureable learning outcomes. Learning outcomes reflect the learning needs analysis, inform and harmonize the training design and development, and are used as a basis for evaluation. A learning outcome is a statement of what the learner is expected to gain – knowledge, skill or attitude - as a result of the learning process.

[1] GOARN Training Programme Concept Note, DRAFT 2, 14 July 2016
[2] Kirkpatrick, Donald L & Kirkpatrick, James D, *Evaluating Training Programmes: The Four Levels (third edition),* Published by Berret-Koehler Publishers, 2006, pg 17.
[3] Swedish Civil Contingencies Agencies (MSB), *Training Materials Development Guide*, Coordination and Operations Department, 2012.

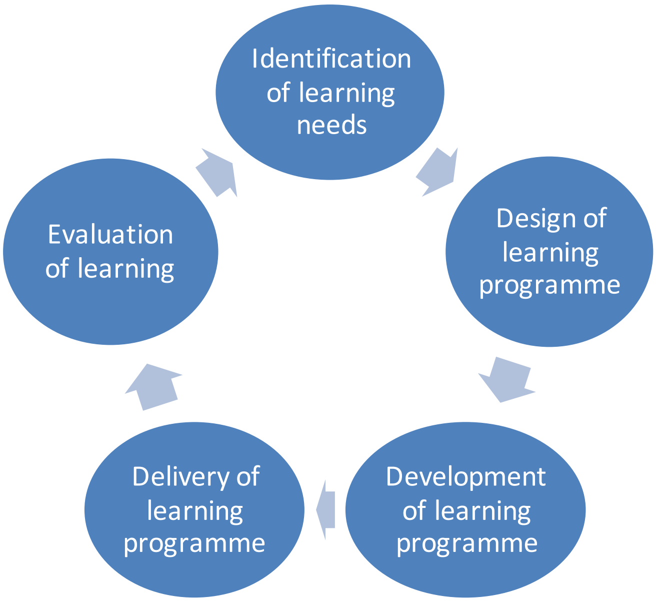


Figure 1. Learning cycle for training and development programmes

It is important to distinguish between a training aim and a learning outcome. An aim, purpose or overall desired outcome of a training course refers to the overall mission that you want to achieve in your course. It is usually a broad statement of what will be achieved and used to concisely describe the course. Learning outcomes are a breakdown of this aim. They are actionable and measurable. It is the sum total of all of these learning outcomes that will contribute to participants meeting the aim of the course.

Learning outcomes should consider the following:

- What results are we trying to accomplish? These results can be stated in such terms as quality, turnover, morale, profits and return on investment (ROI), etc.
- What behaviours are needed to accomplish these desired results?
- What knowledge, skills and attitudes are necessary to achieve the desired behaviours?

The training program curriculum, and subsequent course development, is then based on accomplishing the knowledge, skills and attitudes[4]. **Well written learning outcomes** tell the **trainers what they will teach,** the **participants what they will learn,** and the **observers/donors what will be accomplished in the training programme**.

There are four components of a learning outcome:

1) Audience **(A)** – the learners of the training activity. Often referred to as participant or students.

2) Behaviour **(B)** - what will the audience be able to do as a result of their participation in the training activity? This should be a clearly observable behaviour.

3) Condition **(C)** – the circumstances or context that the learning will occur. This can be a training session, training event, activity, etc.

4) Degree of achievement **(D)** - the standard of which the behaviour will be performed.

[4] Kirkpatrick, Donald L & Kirkpatrick, James D, *Evaluating Training Programmes: The Four Levels (third edition),* Published by Berret-Koehler Publishers, 2006, pg 9.

The **B**ehaviour, or action verb, is the most important element of a learning outcome and can never be omitted, as it states precisely what the participant will be able to do proceeding the instructional activity. These verbs are categorised by domains of learning and various hierarchies, such as Bloom's Taxonomy (see below). Typically, learning outcomes are written in the order of **CABD**. As the **C**ondition and the **A**udience of a training event are usually the same for each session, often the learning outcomes of a session are phrased "At the end of this training session, participants will be able to…", with a list of a few bullet points following that include the measurable verb **B**ehaviour and a corresponding **D**egree of achievement.

Educators and trainers typically use Bloom's Taxonomy to inform the development of curriculum and learning outcomes. Bloom's Taxonomy is a classification system used to define and distinguish different levels of human cognition – i.e thinking, learning and understanding. The original taxonomy was organised into three domains: Cognitive, Affective and Psychomotor. Educators and trainers typically focus on the Cognitive domain, which itself includes six different classification levels: Knowledge, Comprehension, Application, Analysis, Synthesis and Evaluation[5]. Various action verbs are categorised into these classification levels, and are commonly used by learning and training practitioners for selection of an appropriate **B**ehaviour for drafting a learning outcome.

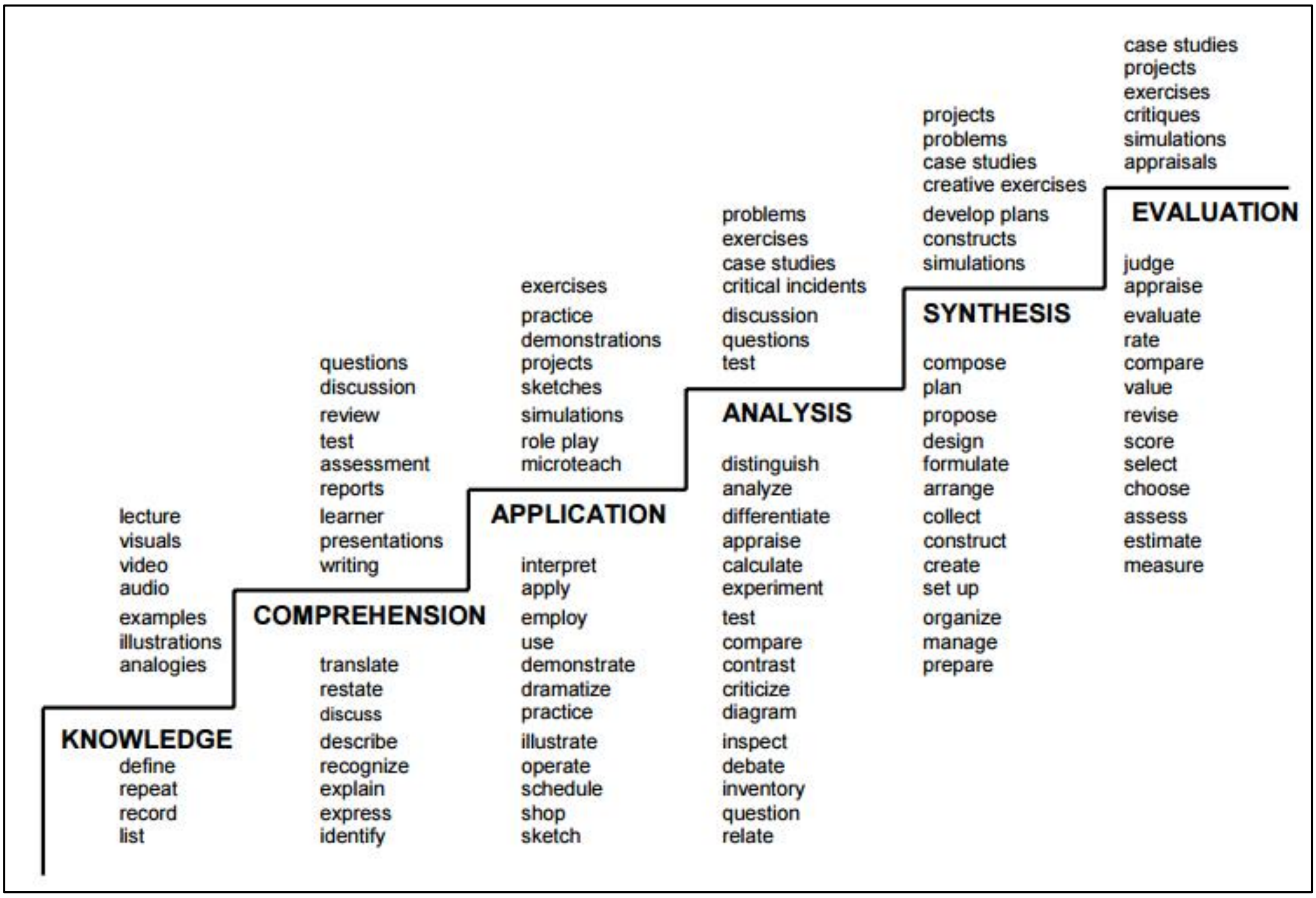


Box 2. Blooms taxonomy staircase[6] with example key verbs for learning outcomes and corresponding suggested teaching and learning activities for measuring its mastery

[5] The Glossary of Education Reform: for journalists, parents and community members, *Bloom's Taxonomy*, http://edglossary.org/blooms-taxonomy/, accessed 31st August 2016.

[6] Cecelia Munzenmaier & Nancy Rubin, Perspectives Bloom's Taxonomy: What's old is new again, pg 16. Accessed 31st August 2016 (original citation from ftp://ftp-fc.sc.egov. usda.gov/NEDC/isd/ taxonomy.pdf )

While designing and developing a training or development programme, it is imperative to consider how you will measure that learning has indeed taken place. I.e. how will you include learning checks to evaluate that the learning outcome has been met? Will this be a test, an assignment or an activity in which participants demonstrate something? Box 2 above illustrates a tool for practitioners to use in drafting learning outcomes and developing subsequent instructional and evaluation activities, according to the cognitive domain of Bloom's taxonomy. The stairs represent the six cognitive levels, arranged in ascending order. Below each step is a list of example verbs that are commonly used to create a learning outcome at that cognitive level, and above each step is a list of suggested activities that can be used to demonstrate and evaluate participants mastery of the learning outcome in question.

The learning outcomes of the various training and development opportunities in the GOARN training programme should be developed to contribute toward building the skills, behaviours and attributes of the behavioural indicators in the GOARN Competency Model for responder team members and team leaders. Mastery of the learning outcomes of each of the tiers of the training programme will combine together to meet the behavioural indicators of the competencies listed in the GOARN Competency Model. Note that it may take completion of more than one tier for participants to demonstrate mastery of a particular competence, with the learning outcomes in each tier contributing in part to the skills/behaviours/attributes of a competency behavioural indicator. For a sample break down of the possible relationship between learning outcomes, behavioural indicator and competency, please refer to Annex I.

## Kirkpatrick Evaluation Model

The **Kirkpatrick Evaluation Model** and its four levels of training evaluation is one of the most widely recognized and utilised methods of evaluating the effectiveness of training programmes[7][8][9]. It was created by Dr. Don Kirkpatrick in the 1950's, and the model is applied before, during and after training to maximize and demonstrate training's value to an organisation. Evaluation Level 1 refers to reaction, Level 2 to Learning, Level 3 to Behaviour, and Level 4 to results. These levels comprise of progressively difficult metrics against which success is evaluated, with Level 4: Results being the most sophisticated. Level 1 is most frequently measured by questionnaire, either at the end of training, or at the end of each day of a training. Level 2 is most frequently measured by pre / post tests, which could be written knowledge based tests, interviews or observations. Level 3 and 4 are most frequently measured by impact surveys, an evaluation tool to measure the extent to which skills and knowledge learned in the program have translated into improved behaviour and the final results that occurred because the participants attended the training program.

Details of the Kirkpatrick 4 levels of evaluation are as follows[10]:

[7] The Training Industry, *Four Levels of Evaluation*, http://www.trainingindustry.com/wiki/entries/four-levels-of-evaluation.aspx accessed 19th June 2016.
[8] Mind Tools: Essential skills for an excellent career, *Kirkpatrick's Four-Level Training Evaluation Model; Analysing Training Effectiveness, https://www.mindtools.com/pages/article/kirkpatrick.htm, accessed 19th June 2016.*
[9] The Kirkpatrick Partners, *The Kirkpatrick Model*, http://www.kirkpatrickpartners.com/OurPhilosophy/TheKirkpatrickModel, accessed 19th June 2016.
[10] [10] Mind Tools: Essential skills for an excellent career, *Kirkpatrick's Four-Level Training Evaluation Model; Analysing Training Effectiveness, https://www.mindtools.com/pages/article/kirkpatrick.htm, accessed 19th June 2016.*

**Level 1. REACTION**

- Measures how participants reacted to the training. Essentially "customer satisfaction".
- Helps to improve for future trainings. Negative reactions reduce the possibility of learning.
- What to measure? Did participants feel the training was worth their time? What were the greatest strengths and weaknesses of the training? Did they feel the venue was suitable and met their needs? Did the training session design and instruction accommodate their personal learning style?
- How to measure? Surveys and Questionnaires, formative assessment and observation during training and verbal feedback. Summative assessment at end of course to determine if participants are pleased with the various course aspects. This type of evaluation is inexpensive and easy to administer using interaction with the participants, paper or online forms.

**Level 2. LEARNING**

- Measures what participants in the training have learned; the degree to which participants acquired the intended knowledge, skills and attitudes as a result of the training.
- What to measure? Use learning outcomes as a starting point. What *knowledge* has been gained? What *skills* were developed or improved? What *attitudes* were changed?
- How to measure? Evaluation methods can take the form of self-assessment or team assessment; they can be informal or formal assessment. Summative assessment to evaluate knowledge, skills and/or attitudes after trainng. If possible measure before and after training to measure the extent of what participants have learned. Evaluation content should be based on learning outcomes. Formative assessments throughout training course for learning checks. Individual pre- and post-training tests for comparisons. Assessment of action based learning such as work-based projects and role-plays. Observations and feedback by peers, managers and instructors.

**Level 3. BEHAVIOUR**

- Measures the extent to which participants have changed their behaviour as a result of the training. Specifically, it looks at how participants *apply* what they have learned in the training, in their work place, and the impact on their performance.
- What to measure? Has the participant expressed a desire to change? If they have had the opportunity, have they put any of their learning to use? How has their performance improved?
- How to measure? Observation and interviews over time (weeks to several months after training), with the participant and possibly their supervisor/subordinates. Note that conditions must be favourable for participants to have the opportunity to actually apply what they have learned in the training, in their workplace. Individual pre- and post-training tests or surveys. Face-to-face interviews. Observations and feedback from others. Focus groups to gather information and share knowledge.

**Level 4. IMPACT**

- Measures how the overall tangilble results that have occurred as a result of the participant attending the training. This can include production, improved work quality, reduced turn over etc. It is importnat to recognise that these results are the very reason for having the training programme in the first place.
- Can be challenging to measure, as need to distinguish between results based purely on their participation in the training, and not other factors. Assumes Level 3 has been measured and met.
- What to measure? Has the effectiveness of the organisation improved as a result of the training. This could be productivty, output, profits, morale, increased quality, etc?
- How to measure? Surveys and Questionnaires, impact assessments of the response assignment, interviews with the former participant / deplyee and possiblky their supervisor and/or requesting entity post mission.

It is the intention for all four levels of the Kirkpatrick Evaluation model to be used to assess both the direct success of the various training programmes proposed in this programme and framework, as well as the impact of the GOARN

trainings on the success of GOARN technical experts deployed on mission. The Level 1, Level 2 and formative Level 3 evaluations of the programmes, as described above, measure the direct reactions, knowledge based learning and immediate behavioural changes from participation in the training programme in question. Taking this a step further in order to measure the impact of the individuals participation in the training programs on the success of their deployment, and consequently the response to the outbreak or public health emergency, formal Level 3 and Level 4 evaluations can be undertaken. Formal level 3 evaluations would take place while GOARN experts are on mission, and after they complete their mission, either through regular follow ups, self-reflections, supervisor feedback, or other means as necessary. Level 4 evaluations could be conducted as part of a greater evaluation of the outbreak or public health emergency response in question, in order to assess the degree to which the targeted outcomes of the mission response occurred as a result of the training. Both formal Level 3 and Level 4 evaluations can be challenging to undertake, as they require the buy-in, commitment and partnership of the various GOARN partners who would be involved in the outbreak or public health emergency response. To ensure quality and relevant training, GOARN and needs to commit to regular evaluations of all training programmes, and the implementation of lesson learned and programme revisions to meet the changing needs of the network.

Details of the recommended monitoring, assessment and evaluation activities for each Tier of the GOARN Training Programme are summarised in Table 1 below.

Document produced by the GOARN Steering Committee Working Group for Training and endorsed by the GOARN Training Network in 2017.

GOARN
Global Outbreak Alert and Response Network

## Table 1. Suggested methods for evaluating each of the Tier 1, 2 and 3 GOARN Training courses, at each of Kirkpatrick's 4 Levels of training evaluation

| LEVEL | Tier 1: Basic Entry-Level | Tier 2: Intermediate-Level | Tier 3: Advanced-Level |
|---|---|---|---|
| **Kirkpatrick Level 1: Reaction** | For **eLearning courses**:<br><br>To assess the level of user satisfaction of an eLearning course, it is recommended to include a short mandatory questionnaire at the end of each course, containing approximately 5-10 questions. For consistency and comparison of level 1 evaluation across eLearning courses, it is further recommended for the same mandatory questionnaire to be embedded within each course.<br><br>The types of information to obtain from this end of eLearning course questionnaire, include the following:<br>• Appropriateness and relevance of the *content*<br>• Perceived usefulness of any *materials* or documents included in the eLearning course<br>• Satisfaction of the *course design* (such as methods, interactivity, duration etc)<br>• *Overall satisfaction* of the course<br>• How will the learner *apply* what they have learned<br><br>**See Annex I for Sample eLearning Course user evaluation.**<br><br>Level 1 eLearning courses should be regularly monitored and used to gauge the degree of interest in accessing and completing the courses and to inform course revisions (such as content, design, additional languages | For **Outbreak Response Scenario Training**:<br><br>There are many types of level 1 evaluation that can be undertaken at a face-to-face training workshop, and it is up to the training team to decide on which will be most relevant and effective. Caution is advised in overdoing evaluation at face-to-face trainings, as too much evaluation after every session, every day and after the training, can tire participants and negatively impact their engagement and skew evaluation results. It is essential to balance and use a variety of methods to evaluate.<br><br>It is recommended for both formative and summative level 1 evaluations to be undertaken. This is open to training content, structure, design, venue, trainers etc.<br><br>Formative evaluations should take place regularly throughout the training course, with the feedback used to adjust the format of the training sessions yet to be undertaken (if possible). Methods for formative evaluation could include the following:<br><br>• Suggestion box / wall<br>• End of session evaluations, which could take the form of informal plenary discussions, or informal chats with participants over coffee breaks etc. Informal conversations are important tools for evaluation as they can identify any problems, needs, and issues early; someone too shy or polite to give formal constructive criticism or feedback might mention something important in a private conversation | For GOARN **Leadership Training**:<br><br>For a tailored GOARN leadership training course, the level 1 evaluation of the training workshop would take same format as described in Tier 2.<br><br>For participation of **GOARN partners in existing Leadership Training Courses**:<br><br>Upon completion of their participation in a leadership training course, a short mandatory questionnaire could be issued to obtain information on their satisfaction of the course, and if they would recommend other GOARN partners to participate. This questionnaire could be of a similar nature to that of Annex II. |

| | | | |
|---|---|---|---|
| | or the creation of new courses on relevant content).<br><br>For the **<u>online hosting platform</u>**:<br><br>To assess user satisfaction with the hosting platform which is used to access and complete the eLearning courses, the follow means of level 1 evaluation can be undertaken:<br><br>Periodic surveys to GOARN registered users on the hosting platform to assess their satisfaction with the hosting platform. The types of information to obtain in these periodic surveys could include the following:<br>• Ease of access to the hosting platform, including registration, logging in, connectivity/bandwidth to platform and courses.<br>• Satisfaction with navigating the platform and accessing the eLearning courses<br>• Interest in / engagement with any Communities of Practice or Discussion Forums<br>• Satisfaction with the content, including relevance and diversity of eLearning course topics and resources/documents, language of content, etc.<br>• *Overall satisfaction* of the hosting platform and it's features<br><br>If there is a Community of Practice or Discussion Forum feature on the hosting platform, it is recommended to include a forum for user questions and concerns. This should be monitored regularly and used as a means of acknowledging and responding to users, and revising | • End of day evaluations, which could include:<br>○ 1-minute feedback forms with a few questions to obtain information on which key elements they learned that they will apply in their work/deployment, questions they have after finishing the days sessions, any other comments.<br>○ Structured format of participants breaking into small working groups and developing 4-5 key feedback points on the day.<br>○ Group debrief sessions with trainers or group mentors.<br>○ +/- charts (pro's and con's lists) : in groups, participants list 2-3 of the most favoured aspects of the day, as well as 2-3 of the least favoured aspects of the day. For further and more detailed feedback, participants can then be instructed to move around room and either place a tick or a cross beside the list items of other groups, to indicate if they agree or disagree.<br><br>The summative evaluation at the end of course needs to take a far more structured and time to achieve its aim. This should include a formal questionnaire/survey, and possibly also a plenary reflection exercise and moderated discussion. The types of information to be collected include<br>• Appropriateness of the *content*<br>• Where the *materials* useful?<br>• Were the *training methods* appropriate/effective?<br>• Were the *trainers/facilitators* effective?<br>• Were participants motivated to learn the contents?<br>• Was the training programme relevant to the participant needs?<br>• Was the time and length of the programme appropriate? | For the GOARN **<u>Development Consultancies</u>**:<br><br>This would entail a combination of formative and summative assessments.<br><br>The formative assessments could include periodic meetings (every couple of weeks), as well as informal conversations over coffee / lunch etc, to assess the consultants satisfaction with the programme and their current available learning opportunities, for possible adjustment.<br><br>At the end of the consultancy, a summative assessment should be undertaken. This could be a debrief session to assess the consultants overall satisfaction and perceived value of the program. This debrief should include a questionnaire (for consistency of analysis and comparison with future development consultants feedback), as well an interview to obtain a more detailed level of feedback and |

| | | | |
|---|---|---|---|
| | the hosting platform as necessary.<br><br>If there are cohorts of users who have completed all available eLearning courses (who have either been deployed or not been deployed), if possible, it is recommended to conduct focus group discussions for detailed feedback on their engagement and opinions on the platform. These discussions could take place via webinar.<br><br>Analysis of the user registration data can also be used to analyse the geographic, language, or technical profile of user engaging with the platform. | • Were the training facilities appropriate?<br>• What is participants overall rating of the programme?<br>• What were the most valued aspects/topics of the training?<br>• How will participants apply what you have learned?<br>• What could be improved?<br><br>**See Annex II for Sample face-to-face training course user evaluation.**<br><br>Note: the same format of level 1 evaluation should be undertaken for any Training of Trainers course for expanding faculty for the Outbreak Response Scenario Training. | recommendations for program improvement. |
| **Kirkpatrick Level 2: Learning** | For **eLearning courses**:<br><br>All eLearning courses should have an embedded mandatory test at the end of the course, and/or learning checks placed periodically throughout the course (for example, at the end of a particular topic).<br><br>If eLearning courses are developed using software such as Articulate Storyline, courses can be designed with these learning checks and end of course tests with questions such as multiple choice, drag and drop, true/false, matching/ordering combination of answers from drop down menus, fill in the blanks, short answer questions (if a moderator will assess answers) and more.<br><br>All learning check and end of course test/quiz questions | For **Outbreak Response Scenario Training**:<br><br>Both formative and summative level 2 evaluations should be undertaken at the face-to-face training.<br><br>The most common form of level 2 training evaluation in terms of improving the knowledge, skills and attitudes of the participants, uses near Identical tests for pre- and post- testing to compare scores before and after the training. This process usually involves identifying what knowledge/skills/attitudes are to be measured for development (based upon learning outcomes), selection of appropriate questions for testing, and the design and development of pre/post-test.<br><br>As this training is primarily a soft-skills development training, the pre/post-tests will need to be designed to measure soft skill progress accordingly. The following pre/post-test level 2 | For GOARN **Leadership Training**:<br><br>For a tailored GOARN leadership training course, the level 2 evaluation of the training workshop would take a similar format to that as described in Tier 2. If technical knowledge regarding GOARN leadership responsibilities and processes are included in the learning outcomes, then a short written knowledge-based pre/post-test could also be used (in addition to the soft skill assessments). |

| | | | |
|---|---|---|---|
| | should reflect the course/module learning outcomes, and only be related to content directly covered in the course. For a 30 minute course, an end of course test of approximately 5-10 questions is sufficient.<br><br>While the exact learning check and end of course test questions will be formulated depending upon the type/s of information you wish to obtain, the following elements should always be considered:<br><br>• Are all learning checks and end of course test questions relevant, challenging, clear and complete?<br>• Are the assessments interactive and engaging, and do they clearly reflect the learning outcomes, and only focus on content that has already been presented to the learner?<br>• Have a variety of testing methods been used throughout, and at the end of the eLearning course?<br>• Is there a pass/fail structure in place? Ie. What percentage of correct answers is required to pass the course? How many times will learners be allowed to take the test? (It will depend upon the purpose of the course, however in general 80% correct responses is required, and learners have no limit to the number of attempts)<br>• Is there a mechanism enabled for feedback to incorrect/correct answers to learning check and end of course questions? | evaluation process is recommended for the outbreak response scenario training:<br>• Collection of pre-training data related to the soft-skill learning outcomes could be undertaken via competency-based interviewing of participant candidates. The selection of competencies and relevant behavioural indicators for assessment will need to be carefully considered according to the feasibility of competency measurement in a phone interview methodology. To ensure transparency and prevent subjective interpretation, assessment criteria for the degree of mastery of each competency assessed will also need to be developed and used in the interview assessment process.<br>• Post-training data collection would then involve assessment of the same competencies and behavioural indicators as the pre-test, using the same assessment criteria, at the end of the training. The formative assessments described below should be largely used to inform this post-assessment, with consideration of the participants progress over the course of the training. A final individual debrief with participants can provide an opportunity for participants to self-reflect on their progress and the identification of any areas still in need of further development.<br><br>Formative evaluations would take place regularly throughout the entire training course as learning checks, most often initiated and assessed by the lead trainer of the session who will assist participants to learn more quickly and more completely by providing them with ongoing constructive feedback. The trainer can also use the feedback from these learning checks to adjust the content and methods of the training to better meet the | For participation of **GOARN partners in existing Leadership Training Courses**:<br><br>GOARN partners participating in an existing leadership training course, should be exposed to level 2 evaluations in the training itself. If possible, GOARN OST could seek to obtain the level 2 evaluation results from the training coordinator of the course. Further, it would be recommended to debrief the participant for their self-reflection on what they learned and their progress towards meeting the learning outcomes, as well any areas they still feel are in need of further development.<br><br>For the GOARN **Development Consultancies**:<br><br>Formative assessments could include feedback from the consultant's supervisor on relevant pieces of work. |

| | | | |
|---|---|---|---|
| | For the **<u>online hosting platform</u>**:<br>If there is a Community of Practice, Discussion Forums and Webinar features enabled for GOARN partner engagement, they can also be used to evaluate learning. For example, if there is webinar open to registered GOARN users on a particular topic, then a discussion forum can be started with a few technical follow up questions for the participants' consideration and answer. These questions would be open-ended and reflect the content of the webinar. The discussion forum would then be monitored to assess the answers, and provide feedback to those learners who have responded to the questions. | participants' needs.<br><br>Specific activities for formative evaluation can be selected according to the learning outcome desired behavioural change (as per Blooms Staircase in Box 2 above), and could include the following elements:<br>• Questions by the lead trainer / facilitator in plenary to participants. These formative questions are used to test the participants understanding on particular content matter (the degree of which will relate to the learning outcome). These questions are not scored, rather the lead trainer/facilitator imparts constructive feedback. These questions can be asked either before or after content is presented, depending upon whether the intent is to obtain learning check information in a pre or post-test manner. In order to assess the progress of learning, these sorts of questions might be asked both before and after content is presented/explored.<br>• Observation of participants in group work and scenario activities. This observation will provide insights into the group dynamics and soft skills of the individual participants. As this is primarily a soft-skills training, this form of observation is important to take regular note of to assess the participants' progress throughout the training. Observation could be undertaken by team mentors and/or the lead trainers of the relevant sessions, and be noted against the session activity learning outcomes.<br>• Hot debrief discussions after scenario activities. This provides an opportunity for the trainer/role player to address any observed issues related to both the content and soft skill competence that the participants have exhibited. If team mentors are also able to continually | At the end of the consultancy, a summative assessment could be undertaken, comparing their deliverables against their Terms of Reference and work plan. Further, it would be recommended to debrief the consultant for their self-reflection on what have they learned and their progress in delivering the relevant pieces of work, as well any areas they still feel are in need of further development. |

| | | | |
|---|---|---|---|
| | | observe both the scenario activities and the debriefs, they can make further assessments of evolving competence (for example, seeking feedback and using constructive criticism to improve performance, taking responsibility for team efforts, etc).<br>• Individual or group presentations. Assessment of participants giving presentations provides insights into both their soft skills of the way in which they present, in addition to their mastery of the technical content they are presenting. Observation and assessment of these skills should be noted against the session activity learning outcomes.<br>• Daily participant self-reflection and mentor-led group discussion. This would provide an opportunity to consolidate assessment and feedback on all of the above formative assessment methodologies, with the mentor summarising daily progress of their group participants. Documentation of this progress is essential for consideration in the summative post-test described above (and can be documented using the pre/post-test assessment criteria as a basis). | |
| **Kirkpatrick Level 3: Behaviour** | For Tier 1 users who are deployed to the field:<br>To evaluate the extent to which Tier 1 users who have completed the mandatory eLearning courses, are applying and benefiting from the new knowledge/skills/attitudes gained from these courses while on deployment, follow up with these users/deployees in needed after, and if possible, during their mission assignment (depending upon the duration of their mission).<br>• Depending upon the communication/reporting | For Tier 2 graduates who are deployed to the field:<br>Similar to Tier 1 graduate deployees, to evaluate the extent to which Tier 2 graduates are applying and benefiting from the new knowledge/skills/attitudes gained from the Outbreak Response Scenario Training Course, follow up with these graduates in required after, and if possible, during their mission assignment (depending upon the duration of their mission). Note: the collection of data for Level 3, should be undertaken concurrently for Tier 1, 2 and if relevant, 3.<br>• Depending upon the communication/reporting operating | For GOARN **Leadership Training**:<br><br>The same evaluation process as Tier 2, with questions tailored to assess the benefits to the role as an effective team leader. In addition to obtaining data from a mission supervisor (if applicable), questionnaires |

| | | | |
|---|---|---|---|
| | operating procedures of the deployee with the GOARN OST during mission, if there is a mid-assignment check in with the deployee, include an agenda item on the relevance and impact of their online pre-deployment training on their mission.<br>• If there is a post-mission debrief, include agenda item to briefly discuss the relevance and impact of their online pre-deployment training on the effectiveness and overall success of their mission. Ask questions regarding the opportunities they have had to apply what they learned in the eLearning courses, and if/how this benefited their deployment.<br>• After the deployee has completed their mission, have them complete a mandatory questionnaire regarding the relevance, applicability and personal impact of the Tier 1 eLearning courses. Include both-closed, scaled and open-ended questions to obtain the following types of information:<br>○ The benefits of the individual eLearning courses on their mission.<br>○ The extent to which their mission environment allowed for the possible application of the skills/knowledge.<br>○ Any topics or processes not covered in Tier 1 that they think should be?<br>○ Did the eLearning courses overall adequately prepare them for deployment?<br>• Obtain feedback from the deployees supervisor on assignment. This could be either through a | procedures of the deployee with the GOARN OST during mission, if there is a mid-assignment check in with the deployee, include an agenda item on the relevance and impact of the Outbreak Response Scenario Training on their mission.<br>• If there is a post-mission debrief, include agenda item to briefly discuss the relevance and impact of their Outbreak Response pre-deployment training on the effectiveness and overall success of their mission. Ask questions regarding the opportunities they have had to apply what they learned in the training course, and if/how this benefited their deployment.<br>• After the deployee has completed their mission, have them complete a mandatory questionnaire regarding the relevance, applicability and personal impact of the Tier 2 Outbreak Response Training. Include both-closed, scaled and open-ended questions to obtain the following types of information:<br>○ The benefits of the Outbreak Response Training on their mission, which topics/elements were most useful etc.<br>○ The extent to which their mission environment allowed for the possible application of the skills/knowledge.<br>○ Any topics or processes not covered in Tier 2 that they think should be?<br>○ Did the Outbreak Response pre-deployment training overall adequately prepare them for deployment?<br>• Obtain feedback from the deployees supervisor on assignment. This could be either through a short interview, or a standard short questionnaire which assesses the extent to which the deployee was | could be given to the subordinates/team members regarding the leadership style and impact on the success of the team.<br><br>For participation of **GOARN partners in existing Leadership Training Courses**:<br><br>This would take the same format as the evaluation for GOARN Leadership Training above, with the intent to assess the relevance and impact of the existing leadership training course on the deployees role as a team lead.<br><br>For the GOARN **Development Consultancies**:<br><br>Periodic follow up with former Development Consultants, and if possible their supervisors, to assess the ways in which their consultancy with GOARN has impacted their job performance.<br><br>3-6 months after the completion of their |

| | | | |
|---|---|---|---|
| | short interview, or a standard short questionnaire which assesses the extent to which the deployee was adequately prepared for the mission, the effectiveness of their integration into an existing team or assignment, knowledge of the outbreak investigation / response architecture, key stakeholders and processes.<br><br>For <u>Tier 1 users who have not been deployed to the field:</u><br>Through periodic surveys, questionnaires or focus group discussions (by Webinar, teleconference or other means), with registered Tier 1 users who have completed 5 or more of the eLearning courses, but have not been deployed, obtain the following types of information:<br>• Reasons for completing the eLearning courses<br>• If they have sought to be deployed to via GOARN, and if so, their thoughts on why they have not been deployed.<br>• If the content of the eLearning courses has been relevant to their ongoing work at their own institutions.<br>• If they have been able to apply the knowledge gained from the eLearning courses in their work, and if so, what has been the benefit.<br>• Include questions related to their interest in and engagement with GOARN, their knowledge of the international public health landscape, other eLearning course topics they think would be beneficial etc. | adequately prepared for the mission, the effectiveness of their integration into an existing team or assignment, knowledge of the outbreak investigation / response architecture, key stakeholders and processes, and the identification of any particular knowledge or skills that were lacking.<br><br>For <u>Tier 2 graduates who have not been deployed to the field:</u><br>Through periodic surveys, questionnaires or focus group discussions (by Webinar, teleconference or other means), with Tier 2 graduates who have not been deployed, obtain the following types of information:<br>• If they have sought to be deployed to via GOARN, and if so, their thoughts on why they have not yet been deployed.<br>• If the content of the Outbreak Response Training has been relevant to their ongoing work at their own institutions.<br>• If they have been able to apply the knowledge gained from the Outbreak Response Training course in their work, and if so, what has been the benefit.<br>• Include questions related to their interest in and engagement with GOARN.<br>. | consultancy, send a questionnaire to obtain information on the ways in which their consultancy experience has benefited / impacted their job performance, and their engagement with GOARN and interest in future deployments. If possible, also conduct an interview or survey with the development consultant's supervisor at their institution, to obtain information on how their experience as a GOARN consultant has impacted their job performance (confidence, productivity, output, interests, attitude, knowledge etc). |

| | |
|---|---|
| **Kirkpatrick Level 4: Impact** | A level 4 evaluation is undertaken to assess the overall impact that the training has had on the organisation, and thus relates here to the impact that the entire GOARN training programme has had on the deployment of experts working in response to outbreak and public health emergencies. This type of evaluation could be conducted as part of a greater evaluation of the outbreak or public health emergency response in question, or a series of outbreak responses over a certain period of time, with focus here to assess the degree to which the targeted outcomes of the mission response/s occurred or did not occur as a result of the training. The level 4 evaluations can provide data against pre-defined benchmarks, with many of the metric data collected in the level 2 and level 3 evaluations above used as indicators of success (see section below on overall GOARN Training Programme Evaluation). New data to be collected in this wider evaluation / lessons learned, directly relevant to the GOARN training programme, would be a similar nature to the Level 3 questionnaires to obtain more information on the skills, behaviours, attributes and overall performance of the GOARN deployees, and the subsequent response interventions, but given to a wider audience for completion including team leader / members, other colleagues, WHO, MoH and other UN/NGO partners, affected populations etc. Detailed analysis of this data would then be undertaken to correlate the relationship between the GOARN training programme and the impact of the response. The frequency and scale of the overall response evaluation (and subsequent degree of training related data collection and analysis), will be largely dependent upon the wider GOARN or WHO operating procedures for response evaluations and lessons learned assessments. As stated above, Level 4 evaluations are big undertakings, and often challenging as they require the commitment and buy-in of the GOARN partners involved coordinating and participating in the outbreak or public health emergency response. |

## Accreditation, equivalence and franchising of GOARN Training

It will be important to undertake regular monitoring and evaluation of GOARN Partner training courses deemed to have "equivalence" to courses in the GOARN Training Program. This is of particular relevance to GOARN partners who have, or are undertaking development of their own mandatory pre-deployment training. The "equivalence" classification process should be undertaken with careful consideration, based upon comparison of learning outcomes, training methodologies and the way in which the courses undertake at least evaluation for Levels 1 and 2, to ensure that learning is evidently tracked. The monitoring and evaluation of these equivalent courses includes the quality assurance of the courses themselves, and well as the record keeping of GOARN deployees having completed a partner pre-deployment training package, rather than the tier 1 basic entry-level and/or tier 2 intermediate levels of the GOARN Training programme. This distinguished data is important for consideration and inclusion in Level 4 evaluations of the GOARN Training Programme.

Clear guidelines for monitoring, evaluation and reporting will need to be included in the GOARN Training Franchising Principles, to ensure that Levels 1 and 2 evaluations are effectively taking place at every franchised training event. This data will need to be shared periodically with the GOARN OST to ensure up to date metrics on the frequency and nature of the franchised training events. Periodic meetings with GOARN partners who are franchising training should also be undertaken to share compare lessons learned and successes and challenges of the training events hosted by different partners, in different locations, with different participant profiles. This feedback should subsequently be used to inform further revision of the GOARN Training courses for continued improvement and enhanced impact.

### Overall GOARN Training Programme Evaluation

The monitoring and evaluation of the overall GOARN Training Programme will be a continuous activity, comprised of the individual training course assessments and evaluations from each of the three tiers (as described above), as well as periodic evaluations of the programme as a whole. It is essential for the consistent and effective knowledge management of all related training data, including personal information of registered users on the learning platform and participants of face-to-face training events, the Level 1 and Level 2 evaluation data from all training courses and events (including data on whether Tier 2 graduates are recommended for deployment or not), and the subsequent Level 3 and (if possible) Level 4 follow up. Depending upon the online platform that is utilised for the Tier 1, there is the possibility to have all of this information stored on the platform. Alternatively, a database would need to be configured and maintained.

In addition to the evaluations of the Tier 1, 2 and 3 training courses as described in Table 1 above, it is also recommended to undertake a periodic formal evaluation of the entire GOARN Training Programme (recommended approximately every 2 years) to assess the perceived value, impact, interoperability and opportunities for expansion or resource enhancement. This could be conducted by the GOARN Training and Development Coordinator (who will be conducting the regular programme monitoring and evaluation), or alternatively by an external independent contractor for an undoubtedly objective perspective. This evaluation would likely be undertaken with network wide surveys, focus groups discussions and select stakeholder interviews.

For the transparency and accountability of the monitoring and evaluation of the development and implementation of the GOARN training programme and its various training courses, it is suggested to employ a logical framework matrix (or logframe). The logical framework approach follows a hierarchical results oriented planning structure and methodology which focuses all project planning elements on the achievement of one project purpose[11] (the overall

[11] European Commission Civil Society Fund in Ethiopia, *Introduction to Monitoring and Evaluation Using the Logical Framework Approach,* 2016.

goal of the GOARN Training). The scope of the GOARN Training Programme goal, outcomes and subsequent outputs, will largely depend upon the broader mission statement and operating procedures of GOARN, and the resources allocated to the training of GOARN partners and the evaluation of outbreak response missions.

Logframe templates are often provided by an implementing organisation or by a donor funding the project in question. At the time of writing this document there was no logframe template or detailed reporting framework provided by WHO, and so a generic logframe structure has been employed by the author as an example (see Annex IV). It is the further intention for this lograme, or the revised logframe that is used for the work planning, monitoring and evaluation of the final design of the GOARN Training Programme, to be a useful tool for the reporting purposes to donors funding GOARN training activities.

## Final Word

Thorough, consistent and transparent monitoring and evaluation of the Tier 1, 2 and 3 training and development courses is essential to the success of the GOARN Training Programme. It is not enough to simply implement a training course and to confirm that participants liked the course. Proper evaluation is the only the way to ensure that learning has actually occurred, that any new found skills/knowledge/behaviours have actually been applied to job performance, and there has been a resultant impact on the organisation. In other words, evaluation to measure these elements is essential to justify the necessary existence of the training course or programme in question. To ensure quality and relevant training, GOARN needs to commit to consistent evaluations of all training programmes, and the implementation of lesson learned and programme revisions to meet the changing needs of the network.

GOARN
Global Outbreak Alert and Response Network

## Glossary of Key Terms

**ABCD Model for learning outcomes:** A Model to help draft learning outcomes, defining the Audience, the Behaviour needed at the end of the session, specify the Conditions under which knowledge will be used, and determine the Degree of achievement needed.

**Articulate Storyline** – Articulate Storyline is an online training software that is used to create interactive self-directed eLearning courses quickly and easily. Storyline is designed as the eLearning authoring tool that can build highly interactive online and mobile courses, with templates, storyboarding, animation, multimedia support, character templates and advanced editing features. It produces SCORM compliant online content packages.

**Behavioural Indicator –** Observable behaviours that individuals employ or exhibit when they are demonstrating a particular competency. There can be many behavioural indicators for each competency.

**Bloom's Taxonomy –** a classification system used to define and distinguish different levels of human cognition – i.e thinking, learning and understanding. Educators typically use Blooms Taxonomy to inform the development of curriculum and learning outcomes. The original taxonomy was organised into three domains: Cognitive, Affective and Psychomotor. Educators and trainers typically focus on the Cognitive domain, which itself includes six different classification levels: Knowledge, Comprehension, Application, Analysis, Synthesis and Evaluation.

**Blended Learning –** Training programme by which learners participate through two different means. This is most commonly delivered via both online and face-to-face learning experiences.

**Classroom learning** – Learning environment in which learners participate in face-to-face instruction in a classroom style setting.

**Closed-ended questions –** Questions that ask respondents to select one or multiple responses from a list. These can be two-option responses, such as yes/no or true/false, or a rating scale or a checklist.

**Community of Practice (CoP)** – Social learning process that occurs when people who have a common interest in a particular subject or area collaborate over an extended period of time, sharing ideas, strategies, determine solutions and build innovations. CoPs are often established virtually, with members communicating and sharing resources via an online source.

**Competency -** The term "competency" refers to a combination of skills, attributes and behaviours that are directly related to successful performance on the job.

**Cornerstone on Demand** – A global leader of cloud-based talent management software solutions. They work with organisations to build Learning Management Systems (LMS) that can administer, deliver and monitor a variety of trainings to accelerate employee performance, foster social learning and support organisational goals.

**Discussion Forum –** A general term for an online bulletin board where individuals can leave and expect to see responses to messages they have left. Discussion forums can be open or restricted to specific user groups, and can be moderated.

**eLearning –** Learning conducted via electronic media. **Self-directed eLearning** refers to digital learning without the presence of a teacher, whereby the content is presented in way that is self-explanatory and learners are to be self-motivated. **Instructor-led eLearning** refers to digital learning that is instructed by a teacher, who interacts with the learner via some means (discussion forums, webinars, written feedback etc).

**Evaluation** – Systematic determination of merit, worth, and significance of something or someone, using criteria against a set of benchmark standards.

**Evaluation Methods** – Evaluation is a methodologically diverse term involving the use of either or both qualitative and quantitative methods, including survey research and questionnaires, case studies, statistical analysis, among others.

**Evaluation of Learning** – The process undertaken to assess that learning has taken place, either through pre and post testing of a training course, participant observation and assessment, or other relevant means.

**Evaluation of Training** – The process of evaluating the success of training. This can include participant feedback on their satisfaction and enjoyment of a training course, evaluation of training faculty, evaluation of training facility, evaluation of training event organisation, and the evaluation of learning.

**Evaluation Tools** – Used to collect data (often referred to as *data collection tools*). Include, and not limited to, questionnaires, surveys, tests, interviews, stakeholder consultations, focus group discussion, observations and performance records.

**Formative Assessment** – a range of formal and informal assessment procedures conducted by educators during the learning process in order to modify teaching and learning activities to improve student or participant attainment.

**Hosting Platform** – This refers to an eLearning Hosting Platform, a web-based space that allows the hosting of and access to a variety of eLearning courses. In general, an eLearning hosting platform with contain a Learning Management System.

**iLearn** - The official WHO hosting and learning platform, which is administered by the WHO Staff Development and Learning team. The iLearn platform is built with Cornerstone on Demand and contains a sophisticated Learning Management System.

**Instructional Design** - Refers to the act of creating an instructional or learning experience which makes the acquisition of knowledge and skills more efficient, effective, and appealing. The instructional designer will ensure the course design is pedagogically sound and that the act of learning itself can be measured. An instructional designer will work with a subject matter experts and technical developers in the development on eLearning and face-to-face training courses.

**Kirkpatrick's 4 Levels of training evaluation** – Kirkpatrick's Levels 1, 2, 3 and 4 refer to the four levels of the Kirkpatrick Evaluation Model, which is one of the most recognized methods of evaluating the effectiveness of training programmes. Evaluation Level 1 refers to reaction, Level 2 to Learning, Level 3 to Behaviour, and Level 4 to results. It was created by Dr. Don Kirkpatrick in the 1950's, and the model is applied before, during and after training to maximize and demonstrate training's value to an organisation.

**Learning Outcomes** – Statements that describe significant and essential learning that participants will or have achieved, and can reliably demonstrate at the end of a course. Essentially, learning outcomes identify what the learner will know or be able to do by the end of a course.

**Learning Management System (LMS)** – Software application for the administration, documentation, tracking, reporting and delivery of eLearning courses or training programmes.

**Logical framework matrix or LogFrame** - A logical framework matrix (or LogFrame) is a tool for improving the planning, implementation, management, monitoring and evaluation of projects. The LogFrame is a way of structuring the main elements in a project and highlighting the logical linkages between them.

**Moderator** – Oversees the communication activity of an online forum. They moderate the interchange of contributors, make decisions regarding content and the direction of threads, structurally organise discussions and materials, and often contribute to discussions and pose questions for consideration.

**Moodle** – A learning platform designed to provide educators, administrators and learners with a single robust, secure and integrate system to create personalised learning environments. It is currently used by some departments of WHO for the administration of their training workshops.

**Open-ended questions –** Questions with unlimited answers, with the question followed by a blank space for response. They give respondents the opportunity to express their own thoughts.

**openHPI –** A platform for massive open online courses, hosted by the German Hasso Plattner Institute (HPI).

**openWHO** - An open access self-directed eLearning platform under development by WHO Pandemic and Epidemic Diseases department, with the contracted services of OpenHPI. It will serve as hosting platform for open eLearning courses.

**Pre/Post Test –** common format for evaluating knowledge-based learning. Identical tests can be pre and post training to compare knowledge scores before and after a training respectively.

**Pedagogy –** The discipline that deals with the theory and practice of education, concerning the study and practice of how to best teach.

**Pilot Testing a Training –** A first opportunity to test run a newly developed or revised training package to evaluate and obtain recommendations for fine-tuning. A good pilot training will enable opportunities to test logistics, relevance, engagement, value and impact, prior to any scale-up or franchising.

**SCORM –** Sharable Content Object Reference Model (SCORM) is a set of technical standards for eLearning software products. SCORM essentially tells programmers how to write their code so that it can “play well” with other eLearning software. Specifically, SCORM governs how online learning content and Learning Management Systems (LMS) communicate with each other.

**Simulation Exercise** – A practice activity that places participants in a simulated situation requiring them to function in the capacity expected of them in a real event. For many organisations, simulation exercises are becoming standard practice during the recruitment process to evaluate behavioural skills, in addition to assessing technical expertise.

**Summative Assessment** – Evaluates student learning at the end of an instructional unit by comparing it against a standard or benchmark.

**Subject Matter Expert** –An individual or a group of people who have the authority of a particular topic. A subject matter expert will work with an instructional designer for the development of face-to-face training and learning materials, and with both an instructional designer and technical developer in the development on eLearning course.

**Technical Developer** – An individual or company that undertakes the actual creation of an eLearning course using various multimedia and software. A technical developer will work with an instructional designer and subject matter expert in the development on eLearning course.

**Tiers -** These refer to the 3 tiers of the proposed GOARN training programme. Tier 1 is the basic entry-level training, Tier 2 is the intermediate-level training and Tier 3 is the advanced-level training.

**Training and Development Coordinator** – person who coordinates the overall training programme, including the design, development, implementation, monitoring, evaluation and maintenance of the training programme as a whole, as well as the individual training courses. Ideally this individual has the pedagogic and technical skills to also instructionally design, technically develop, facilitate and evaluate online, blended and face-to-face learning.

**Training Facilitator –** An individual who engages in the activity of facilitation, helping a group of people understand their common objectives. They contribute structure and process to interactions so groups are able to function effectively, supporting everyone to do their best thinking and practice. As an often content neutral workshop leader, they create an environment where every participant has the opportunity to collaborate, innovate and excel, encouraging full participation. Often a training facilitator will co-facilitate with a subject matter expert trainer to ensure the learning needs and development of all participants is met.

**Training needs analysis –** The process of identifying the gap in employee performance (in this case a deployee) and the existing training (if there is any), and to identify the related new or revised training needs.

**Trainers –** Here the term trainer refers to a subject matter expert who is leading an instructional learning exercise. They are the content technical experts on site to offer targeted technical advice and answer any technical content related questions. Depending upon their former training and facilitation experience, these sessions may be less learner centred and more presentation or lecture oriented. Technical expert trainers will often work with and/or co-facilitate with a qualified training facilitator to ensure the learning needs and development of all participants is met.

**WebEx** - A company that provides on-demand collaboration, online meeting, web conferencing and videoconferencing applications.

**Webinar -** A seminar that is conducted over the internet, with the use of such software as WebEx.

GOARN
Global Outbreak Alert and Response Network

## Key Resources


Cecelia Munzenmaier & Nancy Rubin, Perspectives Bloom's Taxonomy: What's old is new again, pg 16. Accessed 31st August 2016 (original citation from ftp://ftp-fc.sc.egov. usda.gov/NEDC/isd/ taxonomy.pdf)

Department for International Development (DfID), *A DfID practical note: Guidance on using the revised Logical Framework*, January 2011.

European Commission Civil Society Fund in Ethiopia, *Introduction to Monitoring and Evaluation Using the Logical Framework Approach,* 2016.

JICA, *Manual on Training Evaluation*, http://www.jica.go.jp/project/cambodia/0601331/pdf/english/5_TrainingEvaluation.pdf, accessed 3rd August 2016.

Kirkpatrick, Donald L & Kirkpatrick, James D, *Evaluating Training Programmes: The Four Levels (third edition),* Published by Berret-Koehler Publishers, 2006.

Kirkpatrick Partners, The Kirkpatrick Model for training evaluation is a worldwide standard for evaluating the effectiveness of training: http://www.kirkpatrickpartners.com/OurPhilosophy/TheKirkpatrickModel

Mind Tools: Essential skills for an excellent career, *Kirkpatrick's Four-Level Training Evaluation Model; Analysing Training Effectiveness, https://www.mindtools.com/pages/article/kirkpatrick.htm, accessed 19th June 2016.*

The Kirkpatrick Partners, *The Kirkpatrick Model*, http://www.kirkpatrickpartners.com/OurPhilosophy/TheKirkpatrickModel, accessed 19th June 2016.

Online Integrated Library for Personal, Professional and Organizational Development, *Evaluating Training and Results (ROI of Training)*, http://managementhelp.org/training/systematic/ROI-evaluating-training.htm, accessed 12 August 2016.

Swedish Civil Contingencies Agency (MSB), *Training Materials Development Guide*, Coordination and Operations Department, 2012.

The Training Industry, *Four Levels of Evaluation*, http://www.trainingindustry.com/wiki/entries/four-levels-of-evaluation.aspx accessed 19th June 2016.

Document produced by the GOARN Steering Committee Working Group for Training and endorsed by the GOARN Training Network in 2017.

GOARN
Global Outbreak Alert and Response Network

**Annex I: Relationship between competency, behavioural indicators and sample learning outcomes at more than one Tier. Mastery of the learning outcomes at each of the Tiers 1 and 2 below, combine together to demonstrate confidence that the participant will be able to exhibit the relevant behavioural indicator.**

| Competency | Behavioural Indicators | Example Entry-Level Learning Outcomes for Cultural Awareness course (C2.1) | Example Intermediate-Level Learning Outcomes related to Cultural Differences |
|---|---|---|---|
| **6. Respecting and promoting individual and cultural differences** | 6.1 Understands and respects cultural and gender issues and applies this to daily work and decision making | • Identify cultural and gender issues in outbreak and health emergency response | • Examine an outbreak or health emergency response for cultural and gender issues<br>• Draft interventions to mitigate cultural and gender issues as part of outbreak or public health emergency response |
| | 6.2 Relates and works well with people of different cultures, gender and backgrounds | • Recognise common traits between people of different cultures, genders and backgrounds | • Exhibit the ability to work well with people of different cultures, genders and backgrounds in response to an outbreak or public health emergency |
| | 6.3 Examines own behaviour and attitudes to avoid stereotypical responses | • Reflect on one owns behaviour, considering how others could interpret words or actions. | • Respond to people appropriately, without bias, stereotyping or discrimination |
| | 6.4 Considers issues from the perspective of others | | • Demonstrate awareness of the perspectives of others, through both verbal and non-verbal actions |
| | 6.5 Draws on diversity of skills, backgrounds and knowledge of people to achieve more effective results | • Explain the benefits of combining the diversity of skills, backgrounds and knowledge of people to achieve more effective results | • Examine an outbreak or public health emergency response for the diversity of skills, backgrounds and knowledge of all relevant stakeholders, including the affected population<br>• Formulate interventions to outbreak and public health emergency response that draw upon the diversity of skills, backgrounds and knowledge of relevant stakeholders |
| | 6.6 Establishes and maintains productive partnerships with clients by gaining their trust and respect | | • Endeavour to establish and maintain productive partnerships by gaining trust and respect |
| | 6.7 Takes into account the needs, skills, capacities, and experiences of crisis-affected people and apply these in the response | • Recognise the needs, skills, capacities and experiences of crisis-affected people | • Build interventions for outbreak and public health emergency response upon the needs, skills, capacities and experiences of crisis-affected people |

Document produced by the GOARN Steering Committee Working Group for Training and endorsed by the GOARN Training Network in 2017.

GOARN
Global Outbreak Alert and Response Network

**Annex II: Sample LEVEL 1 evaluation for a self-directed eLearning Course**

| Question | Responses: Multiple Choice box selection *or* Open-ended |
|---|---|
| Please select your reason for undertaking this eLearning module. | ☐ Pre-deployment requirement for GOARN Mission<br>☐ Pre-course work for GOARN Outbreak Response Training<br>☐ General Interest |
| How did you find out about this training? | ☐ Workplace / Supervisor<br>☐ Colleague/Friend<br>☐ GOARN communications<br>☐ Other |
| On a scale of 1 (very low) to 5 (very high), how satisfied were you with the information in this eLearning module? | ☐ 1<br>☐ 2<br>☐ 3<br>☐ 4<br>☐ 5 |
| On a scale of 1 (very difficult) to 5 (very easy), how easy did you find this course to navigate? | ☐ 1<br>☐ 2<br>☐ 3<br>☐ 4<br>☐ 5 |
| On a scale of 1 (very low) to 5 (very high), how likely are you to recommend this eLearning module to a friend or colleague? | ☐ 1<br>☐ 2<br>☐ 3<br>☐ 4<br>☐ 5 |
| What part of this course do you feel was the most relevant for you and why? | |
| How will you use this information gained in either your<br>i. GOARN response mission;<br>ii. participation in GOARN Outbreak Response Training, or<br>iii. daily work | |
| How would you improve this course? | |

Document produced by the GOARN Steering Committee Working Group for Training and endorsed by the GOARN Training Network in 2017.

GOARN
Global Outbreak Alert and Response Network

## Annex III: Sample LEVEL 1 evaluation for a face-to-face Instructor Led Training Workshop

### COURSE EVALUATION FORM

Please answer the following questions based on your own experience in the training workshop. Your responses are an important part of the continuous improvement process of these learning materials and your feedback will help to improve the learning experience for future workshop participants. All responses are confidential and anonymous. Thank you very much for completing this workshop evaluation form.

**PARTICIPANT'S NAME:** ________________________________________ **DATE:** ___________________________

1. How do you evaluate this training workshop overall?
   - ☐ Excellent
   - ☐ Very Good
   - ☐ Good
   - ☐ Poor
   - ☐ Terrible

2. Select the two training workshop topics that will be most useful to you on deployment:
   - ☐ Topic 1
   - ☐ Topic 2
   - ☐ Topic 3
   - ☐ Topic 4
   - ☐ Topic …X

Please indicate the extent to which you agree or disagree with the following statements:

| | Agree strongly | Agree somewhat | Neither agree not disagree | Disagree Somewhat | Disagree Strongly |
|---|---|---|---|---|---|
| 3. The stated outcomes were achieved during the training. | ☐ | ☐ | ☐ | ☐ | ☐ |
| 4. My personal objectives for attending were achieved in the training. | ☐ | ☐ | ☐ | ☐ | ☐ |
| 5. What I learned in this training will benefit me on deployment. | ☐ | ☐ | ☐ | ☐ | ☐ |
| 6. The training content was relevant and challenging. | ☐ | ☐ | ☐ | ☐ | ☐ |
| 7. The training methodology was effective. | ☐ | ☐ | ☐ | ☐ | ☐ |
| 8. The length of this workshop was just | ☐ | ☐ | ☐ | ☐ | ☐ |

right.

9. What parts of this training were most interesting and useful for you? Please explain.

10. How will you apply or integrate this into your work, if deployed?

11. What parts of the training were of little or no value for you? Please explain.

12. In what ways can this training workshop be most improved?

13. Any other comments

Document produced by the GOARN Steering Committee Working Group for Training and endorsed by the GOARN Training Network in 2017.

GOARN
Global Outbreak Alert and Response Network

**Annex IV: Example logframe for the monitoring, evaluation and reporting on the GOARN Training Programme, over a 24 month period.**

Note this example is only related to accessibility, satisfaction and learning of the training courses. This would need to be expanded to include faculty, training of trainer programmes, quality assurance of franchised training courses, network contribution to training etc.

| | Description | Indicators | Means for verification | Baseline/s | Target/s |
|---|---|---|---|---|---|
| **Overall Goal** | Provide quality training for GOARN partners to ensure the provision of effective technical support to WHO Member States experiencing a human health emergency | • # and type of training courses available for GOARN partners<br>• # of level 3 evaluation reports indicating benefits of on the job application of knowledge, skills, behaviours acquired from training<br>• # of level 4 evaluation reports indicating direct impact of training on response missions | • Catalogue of GOARN Training courses<br>• Level 3 evaluation data and reports<br>• Level 4 evaluation data and reports | • 1 course (Outbreak Response Scenario Training)<br>• 0 Level 3 evaluation reports<br>• 0 Level 4 evaluation reports | • 9+ courses (Outbreak Response Scenario Course, Advanced Leadership Training, 7 online basic-entry level courses)<br>• Four Level 3 evaluation data and reports<br>• One Level 4 evaluation data and reports |
| **Outcome #1** | GOARN partners have access to relevant quality training and development opportunities | • # and type of training courses available for GOARN partners<br>• % of GOARN partners who express knowledge of available training opportunities<br>• % of GOARN partners participating in GOARN training courses, expressing high level of satisfaction with the course/s | • Catalogue of GOARN Training courses<br>• Periodic survey to GOARN partners with yes/no question on their knowledge of the individual training course opportunities<br>• Level 1 course evaluations | • 1 course (Outbreak Response Scenario Training)<br>• TBC (will be set based on results of first survey that will be sent out)<br>• TBC (will be set based on the first cohorts of Level 1 evaluations completed) | • 9+ courses (Outbreak Response Scenario Course, Advanced Leadership Training, 7 online basic-entry level courses)<br>• 80% of survey respondents express knowledge of available courses<br>• 80% of Level 1 evaluations scoring highly satisfied with overall course |
| **Output #1.1** | All GOARN partners have access to relevant and quality | • # of Hosting platforms live and accessible | • Hosting platform is developed and gone live | • 0 hosting platforms | • 1 hosting platform |

| | | | | | |
|---|---|---|---|---|---|
| | online basic entry-level courses | • # of registered users on hosting platform<br>• # of eLearning courses accessible<br>• # of users accessing eLearning courses<br>• % of users completing eLearning courses<br>• # of available languages of courses<br>• % of GOARN partners participating in GOARN training courses, expressing high level of satisfaction with the course/s | • Hosting platform registration data<br>• eLearning courses uploaded to hosting platform<br>• Hosting platform analytics (users commencing course)<br>• Hosting platform analytics (users completing courses)<br>• eLearning courses uploaded to hosting platform languages other than English<br>• Level 1 eLearning course evaluations | • 0 registered users<br>• 0 eLearning courses uploaded to the hosting platform<br>• 0 users accessing eLearning courses<br>• 0% users completing eLearning courses<br>• 0 eLearning courses available in at least one language other than English<br>• TBC (will be set based on the first cohorts of Level 1 evaluations completed) | • 1,500 registered users<br>• 7+ eLearning courses on hosting platform<br>• 1,200 users access eLearning courses (80% of registered users)<br>• 80% of users starting courses, complete them<br>• 7+ eLearning courses available in at least one language other than English<br>• 80% of Level 1 evaluations scoring highly satisfied with overall course |
| **Output #1.2** | Select GOARN partners have opportunity to participate in quality and relevant Tier 2 Outbreak Response Scenario Training | • # of individuals from GOARN partner institute apply for participation<br>• # of individuals from GOARN partner institutes participating in training<br>• % of GOARN partners participating in GOARN training courses, expressing high level of satisfaction with the course/s<br>• # of GOARN Outbreak Response Trainings implemented | • Participant candidate application records<br>• Participant records from training courses<br>• Level 1 Outbreak Response Training evaluations<br>• Records from GOARN OST implemented trainings, as well as franchised trainings implemented<br>• GOARN Outbreak Response | • TBC (needs to be consolidated from all previous trainings)<br>• TBC (needs to be consolidated from all previous trainings)<br>• Level 1 Outbreak Response Training evaluations<br>• Records from GOARN OST implemented trainings, as well as franchised trainings | • 50% increase per annum in applications<br>• 50% increase per annum in participation<br>• 80% of Level 1 evaluations scoring highly satisfied with overall course<br>• 50% increase per annum in the number of trainings implemented<br>• Courses available in at least one language other |

| | | | | | |
|---|---|---|---|---|---|
| | | • # of available languages of courses | Training packages | implemented<br>• GOARN Outbreak Response Training packages | than English |
| **Output #1.3** | Select GOARN partners have opportunity to participate in quality and relevant Tier 3 Leadership Training | • # of individuals from GOARN partner institute apply for participation<br>• # of individuals from GOARN partner institutes participating in training<br>• % of GOARN partners participating in GOARN training courses, expressing high level of satisfaction with the course/s<br>• # of GOARN Leadership Trainings implemented<br>• # of GOARN partners participating in existing leadership trainings | • Participant candidate application records<br>• Participant records from training courses<br>• Level 1 Outbreak Response Training evaluations<br>• Records from GOARN OST implemented trainings<br>• Records from GOARN OST on GOARN partner participation in existing available training | • 0 applications<br>• 0 participants<br>• Level 1 Outbreak Response Training evaluations<br>• Records from GOARN OST implemented trainings | • 50% increase per annum in applications<br>• 50% increase per annum in participation<br>• 80% of Level 1 evaluations scoring highly satisfied with overall course<br>• 50% increase per annum in the number of trainings implemented<br>• 50% increase per annum in participation of GOARN partners in existing leadership training |
| **Outcome #2** | Graduates of GOARN training courses benefit from their training participation, while on deployment. | • % of GOARN training course graduates expressing personal and professional benefits from the application of the knowledge, skills and behaviours while on deployment<br>• % of deployees supervisors expressing observed benefits of the deployee having participated in GOARN Training | • Level 3 evaluations | • TBC (will be set based on results of first Level 3 evaluations) | • 80% of graduates expressing benefits of applying knowledge and skills from training while on deployment<br>• 80% of deployee supervisors expressing benefits of the deployee having participating in GOARN training |

GOARN
Global Outbreak Alert and Response Network

| | | | | | |
|---|---|---|---|---|---|
| **Output #2.1** | GOARN partners graduating the Tier 1, Tier 2 and Tier 3 training courses demonstrate an increase in desired knowledge, skills and behaviours after participation in GOARN Training | • % of individuals passing the Tier 1 basic entry level eLearning courses<br>• % of individuals passing the Tier 2 Intermediate Outbreak Response Scenario Training<br>• % of individuals passing the Tier 3 Advanced Leadership Training | • Level 2 evaluations | • 0 Tier 1 graduates<br>• TBC Tier 2 graduates (needs to be consolidated from all previous trainings)<br>• TBC Tier 3 graduates (needs to be consolidated from all previous existing leadership trainings) | • 50% increase per annum in graduates |
| **Output #2.2** | GOARN partners graduating the Tier 1, Tier 2 and Tier 3 training courses, expressed the desire to apply the new knowledge, skills and behaviours acquired after participation in GOARN Training while on deployment | • % of individuals who have passed the Tier 1, 2 and 3 have nominated themselves for deployment<br>• % of selected individuals for deployment who have passed the Tier 1, 2 or 3 courses<br>• % of individuals who have deployed express they had the opportunity to apply what they learned in the GOARN Tier 1, 2 or 3 training/s | • Hosting platform analytics for eLearning graduates<br>• Participant records from Tier 2 and Tier 3 training courses<br>• Records of nominations for deployment<br>• Level 3 evaluations | 0% of graduates nominating themselves for deployment<br>• 0% Tier 1 and Tier 3 graduates being deployed<br>• TBC Tier 2 graduates (needs to be consolidated from all previous trainings)<br>• TBC individuals expressing they have applied what they learned in training courses (will be set based on results of first Level 3 evaluations) | • 80% of Tier 1, 2 and 3 graduates nominating themselves for deployment<br>• 80% of all deployees have graduated from Tier 1, 2 or 3 training courses<br>• 80% deployees expressing that they have had the opportunity and desire to apply what they learned in the training courses while on deployment |
